# Occurrence of *Mycoplasma gallisepticum* in wild birds: A systematic review and meta-analysis

**DOI:** 10.1371/journal.pone.0231545

**Published:** 2020-04-16

**Authors:** Anna Sawicka, Maciej Durkalec, Grzegorz Tomczyk, Olimpia Kursa

**Affiliations:** 1 Department of Poultry Diseases, National Veterinary Research Institute, Puławy, Poland; 2 Department of Pharmacology and Toxicology, National Veterinary Research Institute, Puławy, Poland; Panstwowy Instytut Weterynaryjny - Panstwowy Instytut Badawczy w Pulawach, POLAND

## Abstract

*Mycoplasma gallisepticum* is one of the most important poultry pathogens that can also infect wild birds, but knowledge of potential non-poultry hosts that could be reservoirs of *M*. *gallisepticum* is limited. For the paper presented here, we screened three databases (PubMed, Scopus, and the Web of Knowledge) to find articles on the occurrence of *M*. *gallisepticum* in different wild bird species that were published between 1951 and 2018. Among 314 studies found, we selected and included 50 original articles that met the pre-established criteria. From those publications we extracted the following information: name of the first author, year of publication, year of sample isolation, country, region, number of birds sampled, number of birds tested by each method, number of positive samples, diagnostic criteria, and if birds were wild or captive. Because different detection techniques were used to confirm the presence of *M*. *gallisepticum* in one animal, we decided to perform the meta analyses separately for each method. The estimated prevalence of *M*. *gallisepticum* in wild birds was different by each method of detection. Our summary revealed that *M*. *gallisepticum* was present in 56 species of bird belonging to 11 different orders, of which 21 species were reported suffering both past and current infection. Our work provides information on wild bird species that could be considered potential reservoirs or carriers of *M*. *gallisepticum* and could be helpful to set the direction for future research on the spread and phylogeny of *M*. *gallisepticum* in different hosts.

## 1. Introduction

Mycoplasmas are the smallest self-replicating bacteria that can cause acute and chronic diseases in humans, animals, insects, and plants [1]. More than twenty species of *Mycoplasma* genus have been described in avian hosts, but *Mycoplasma gallisepticum* (MG) is one of the most important pathogens of poultry and wild birds [2]. Mycoplasmosis was described as a respiratory disease of poultry for the first time in the early 1900s. However, there is a discrepancy about when exactly mycoplasmosis was first described. According to Charlton et al. [3], mycoplasmosis was described for the first time as a respiratory disease in turkeys in 1926, and in chickens in 1936, whereas Luttrell and Fisher [4] described its occurrence primarily in domestic poultry in 1905 and Saadh and Hasani [5] defined the first isolation as having been in chickens in 1931 [6]. The causative agent MG was successfully cultured in 1960 by Edward and Kanarek [7]. Since frequent occurrences of MG in flocks of domestic poultry have been being reported, the role of wild birds as potential reservoir and vectors of MG has been of interest to the scientific community worldwide. Initial research in this field focused primarily on MG seroprevalence in the wild turkey (*Meleagris gallopavo*) [8,9]. The first case of MG isolation from the Eurasian tree sparrow (*Passer montanus*) was described by Shimizu et al. [10] in Japan. The authors obtained *Mycoplasma* strains from live tree sparrows caught in chicken pens or captured in the field or from dead birds and MG isolates were identified serologically. Later, in the USA MG strains were also identified in wild turkeys by Jessup et al. [11] and Adrian [12]. However, in all these cases the reason for infection was probably the close contact between wild birds and domestic poultry. One of the most extensively documented MG infections was a large-scale epidemic of mycoplasmal conjunctivitis caused by MG in house finches. Ley et al. [13] was one of the first to successfully isolate MG from house finches with conjunctivitis. Luttrell et al. [14] compared the prevalence of MG in house finches with and without conjunctivitis and the results of the study showed that MG was isolatable from birds with conjunctivitis as well as from healthy birds. Within a few years of the outbreak, the epidemic had spread rapidly across the eastern North American range of the host species [15]. Clinical MG infection was also reported also in American goldfinch (*Spinus tristis*) [16–20], purple finch (*Carpodacus purpureus*), and house sparrow (*Passer domesticus*) [19]. However, the mycoplasmosis did not lead to such large changes in the population dynamics of those species as those reported in house finches [21,22]. Phylogenetic analysis revealed that the source of MG infection in house finches were strains originating from turkeys and chickens [23], and the responsibility for this shift to a novel host may lie with the tuning of the existing gene repertoire of MG that encodes variable antigenic lipoproteins [24]. A more recent experiment showed that domestic poultry could remain susceptible to infection with house finch MG but the virulence of reintroduced strains could be lower than in the house finch [25]. The authors suggested that the virulence of MG strains could decrease with its adaptation to wild species of birds.

In recent decades many methods have been described and adapted for the diagnosis of MG. The available methods are based primarily on culture or molecular and serological tests. The culture method was generally regarded as the gold standard for the definitive diagnosis of mycoplasmal infections. Nevertheless, cultivation techniques have also some weaknesses. The most common problems with the cultivation of MG from a clinical sample in the laboratory are overgrowth of faster-growing *Mycoplasma* species or no growth in the subculture [26]. However, some past studies showed problems with primary isolations of MG in wild birds [13,27]. Also, the species identity for mycoplasmas growing on agar should be confirmed by additional procedures, e.g., by polymerase chain reaction (PCR) or immunoserological assay [28].

The development of new laboratory methods has had a great impact on the diagnosis of clinical infections, delivering results in much less time. The primary advantage of PCR is that it is a rapid and sensitive method of detection of DNA which provides an alternative method for direct detection of the organism. Additionally, it represents a useful tool for molecular characterization and may help in epidemiological studies to determine the source of infections and the relationships among strains.

Different serological assays were used to diagnose MG, including the serum plate agglutination assay (SPA), hemagglutination inhibition test (HI), and enzyme-linked immunosorbent assay (ELISA). The SPA test is quick, relatively inexpensive, and sufficiently sensitive and it has been commonly used as a screening procedure in routine programs of MG infection monitoring in commercial poultry. Antibodies against MG can be detected a minimum of one week after infection in agglutination tests and up to three weeks by HI [29]. The SPA assay detects immunoglobulin M (IgM), which is an indicator of recent primary infection. Due to nonspecific reactions and interspecies cross-reactivity, all of the positive results detected by the SPA should be confirmed by a different method. The HI assay might be expected to be confirmatory, but in fact, it detects only the Y class of immunoglobulins (IgY), often mislabeled as immunoglobulin G (IgG). Immunoglobulin Y is responsible for responding to mycoplasma antigen in later stages of infection [30], and therefore detecting both types of immunoglobulins at the same time is sometimes impossible.

The ELISA is another serological assay measuring antibody levels. However, it is rarely used for the diagnosis of *Mycoplasmatales* in wild birds. The main problem with using ELISA is the lack of commercial antibodies for detection of immunoglobulins from wild birds [31]. Previous studies have shown that an antibody that was produced using sera of four species recognized multiple avian species and provided breadth of coverage for bird diagnosis [32]. Anti-chicken antibodies have also been determined by Martínez [31] as useful for different bird species. Nevertheless, each of those solutions has some limitations and any positive results obtained using a broad-spectrum secondary antibody should be confirmed by another method [33].

A synthetic summary of the previous work on *Mycoplasma gallisepticum* in various species of wild birds could be a valuable source of information on the occurrence of this pathogen in different hosts worldwide. Our study aimed to evaluate and summarize the existing knowledge on the occurrence of MG in wild birds originating from different countries located in different parts of the world, belonging to different orders and species, and categorized as wild or captive (if such information was available). In our work, we revealed numerous wild bird species that could be considered as potential reservoirs and carriers of MG. We also synthesized the information on the most common methods and techniques used for the detection of MG infections to find potential gaps in procedures and ways of reporting the results.

## 2. Materials and methods

Systematic review and meta-analysis were used to estimate *Mycoplasma gallisepticum* occurrence in wild birds to identify data gaps. Our work was performed following the preferred reporting items for systematic reviews and meta-analyses (PRISMA) statement [34] and the meta-analysis of observational studies in epidemiology (MOOSE) guideline [35]. The PRISMA checklist is reproduced in S1 Table.

### 2.1. Literature search

Literature searches for published studies were made during the first week of January 2019. Three databases (the Web of Science, PubMed, and Scopus) were searched for studies published from the 1st of January, 1951 to the 31st of December, 2018 using the conjunction of the following key words: “*Mycoplasma gallisepticum*”, “wild”, “birds”. We also screened the reference lists of all retrieved articles and previous reviews by Benskin et al. [36], Dhondt et al. [2], and Faustino et al. [37] to find other relevant publications by hand.

### 2.2 Selection criteria

The first selection of studies was made based on information specified in the title and abstract. The second step was to verify the full text of the article, if available. Studies were included if they met the pre-established criteria: (1) they reported data from an original peer-reviewed study; (2) they contained extractable information about the occurrence of MG in wild bird species (3); they provided an adequate description of the bird species tested (4); they provided a sufficient description of the method used (5); they defined the outcome of serological, culture or molecular biology methods (6); and they were published in English. Studies providing a review or reporting genomics, duplicate publications of the same study, articles available only as an abstract, and experimental trials were excluded. However, we included data from two articles [14,18] in which birds were captured in the wild, tested for the presence of MG, and then used in experimental trials. Only data for MG screening after capturing of birds were extracted from those two papers and included in our database. We also excluded articles or parts of articles in which the occurrence of MG was detected in semi-wild birds, pet birds or birds kept in zoological gardens.

### 2.3. Data extraction

Full texts of articles were reviewed, and relevant data were extracted independently by two authors (AS and MD). In the case of disagreements, all of the concerns were resolved by discussion. The following information was extracted from each study: the author’s name, year of publication, year of study, country, region, number of birds sampled, number of birds tested by each method, number of MG-positive samples, and diagnostic criteria. The number of birds tested and the number of samples found positive by the culture method and PCR were extracted regardless of the part of the respiratory tract from which the swab was taken. All extracted records were also categorized according to the birds’ habitat as “wild”, “captive” or, if that information was not available in the text, “unknown”. Data were extracted and coded into a predefined table using Excel (Microsoft Office 2016, Microsoft Corp., Redmond, WA, USA). The created database was double-checked independently by two authors (AS and MD).

### 2.4. Data preprocessing

Because we found inconsistencies in the taxonomic nomenclature used by authors, we unified scientific and common names of bird species according to the current taxonomic nomenclature (Avibase, n.d.) and we limited the level of detail to the species. We also unified the names of diagnostic tests (e.g. rapid serum plate agglutination (RSA), serum plate agglutination (SPA), and plate agglutination tests (PA) were coded as “SPA”).

### 2.5. Bias assessment

It was found that conventional funnel plots (plots of the log of effect measures versus standard error) are inaccurate in analysis of publication bias of proportional studies because they produce spurious asymmetry in the plot even when publication bias does not exist [38]. Due to the high diversity between study designs, unequal and small sample sizes, the presence of zero events in some selected papers and the limited application of publication bias assessment in studies of the prevalence, we decided to include all of the papers without assessing the bias of individual studies.

### 2.6. Statistical analysis

All calculations were performed using R version 3.6.1 [39]. The *dplyr* package version 0.8.3 was used for data manipulation [40]. We performed a meta-analysis of single-proportion data using the inverse-variance method and arcsine transformation, which appears to be the best classic method of data transformation for this type of data [41]. The estimated prevalence and 95% confidence intervals (CIs) were calculated using the *metaprop* command implemented in the *meta* package version 4.9–7 [42] after the choice was made of the Wilson method was for the calculation of the intervals [43]. For the pooled data, an inconsistency index (I^2^) was calculated to estimate the heterogeneity. The random-effect model was chosen because of high heterogeneity. We also performed a subgroup analysis for countries, regions, orders, species, and captivity status. Because in many articles more than one diagnostic method was reported, we decided to perform meta-analyses for each method separately. The following packages were used to visualize search results: *ggplot2* version 3.2.1 [44], *wordcloud* package version 2.6 [45], and *upset* package version 1.4.0 [46].

## 3. Results

### 3.1. Search summary

**Fig 1:**
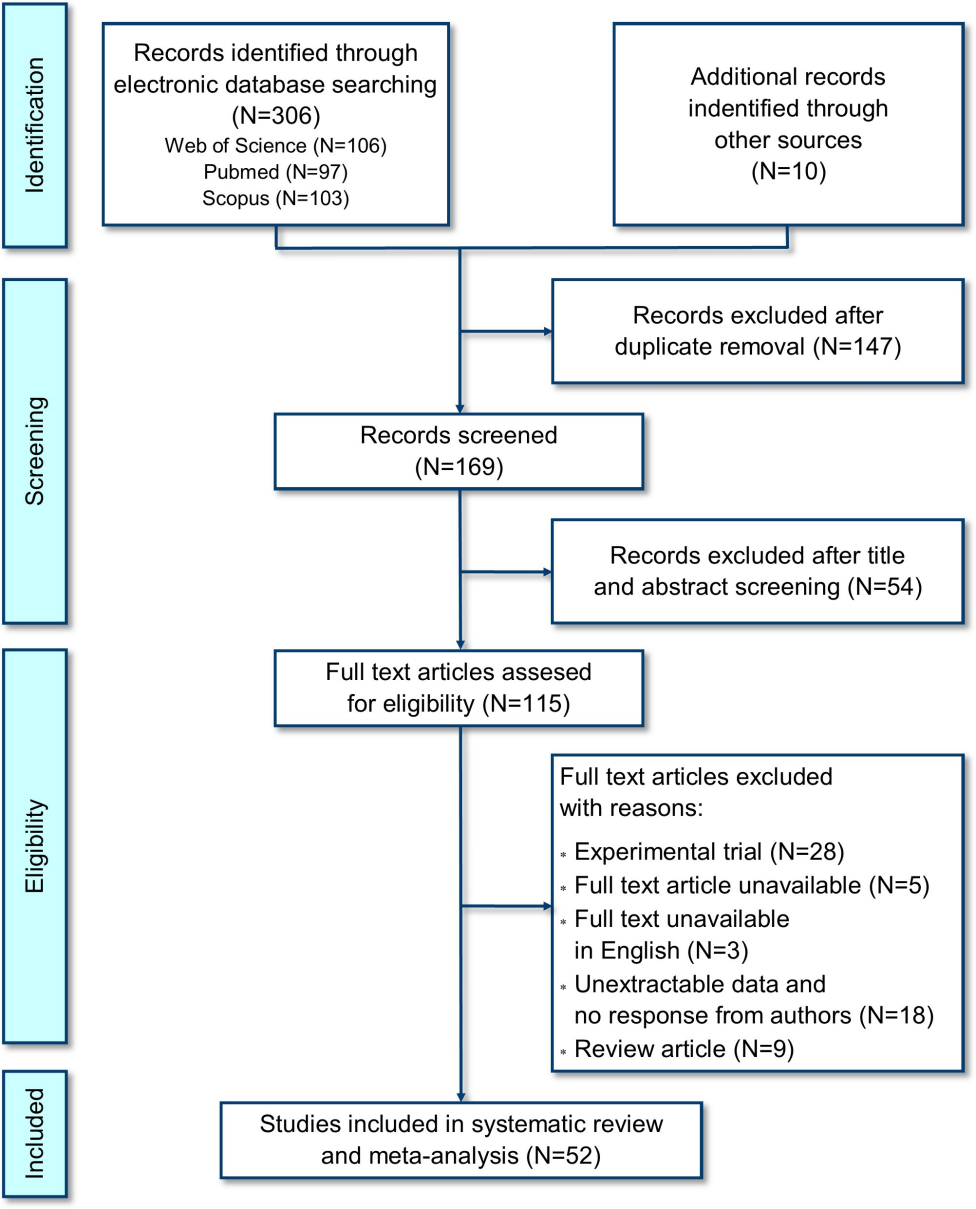
Flow diagram. The diagram shows the number of studies on the occurrence of *Mycoplasma gallisepticum* in wild birds from 1951 to 2018 that were found, assessed, included and excluded.

A total of 316 studies were collected from databases and by hand searching. Articles totaling 147 articles were removed as duplicates. After the first assessment based on the title and abstract evaluation, 54 articles were excluded, yielding 115 studies for the second screening that seemed eligible for full-text review. There were six articles among those papers that did not contain all of the necessary information. We contacted the corresponding authors and we did not receive any reply, and consequently those articles were excluded at that stage. In summary, a total of 63 papers were excluded after assessment of the full text because they not met the pre-established criteria. A total of 52 papers were included based on the results of the systematic review, as shown in Fig 1 and listed in S2 Table. Data extracted from publications and included in systematic review and meta-analysis are available in S1 Dataset.

Of these, 35 of the papers were from the USA, 4 were from Brazil, 2 were from the Galápagos Islands, 4 were from European countries, 3 were from other North America countries, 3 were from Asian countries and 1 was from Namibia and South Africa (S3 Table).

**Fig 2:**
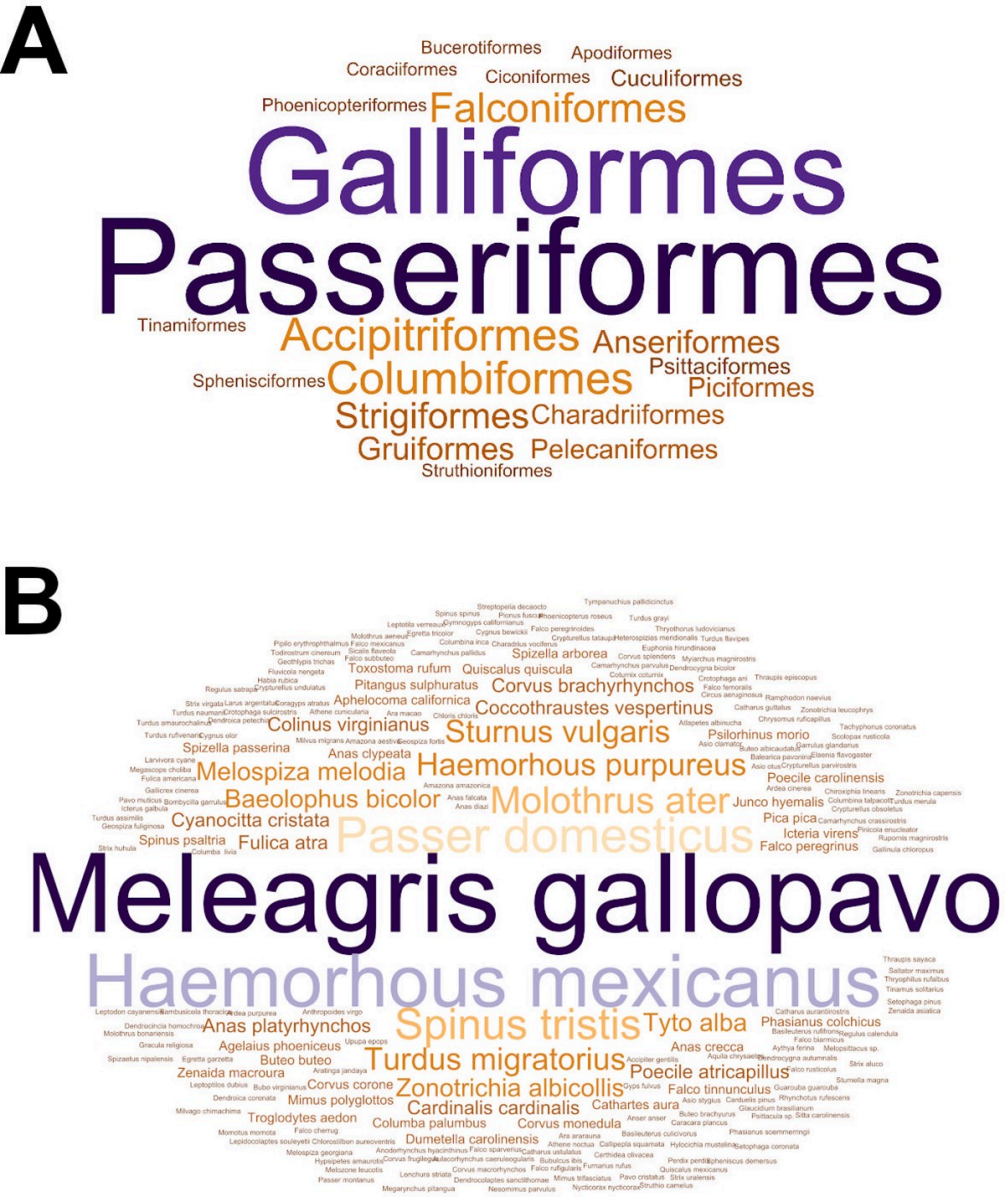
Word clouds showing the number of studies on the occurrence of *Mycoplasma gallisepticum* in wild birds from 1951 to 2018 that were found by order (A) and by species (B). The size of the word indicates the number of studies concerning particular order or species.

The majority of the studies concerned *Passeriformes* (N = 26) and *Galliformes* (N = 23) followed by the *Columbiformes* (N = 7), *Accipitriformes* (N = 7), and *Falconiformes* (N = 6), *Anseriformes* (N = 4) (Fig 2A) and the most frequently tested species were the wild turkey (N = 18) and house finch (N = 12) (Fig 2B). For the clarification, the number of studies by order and species of birds was summarized in S4 Table.

**Fig 3:**
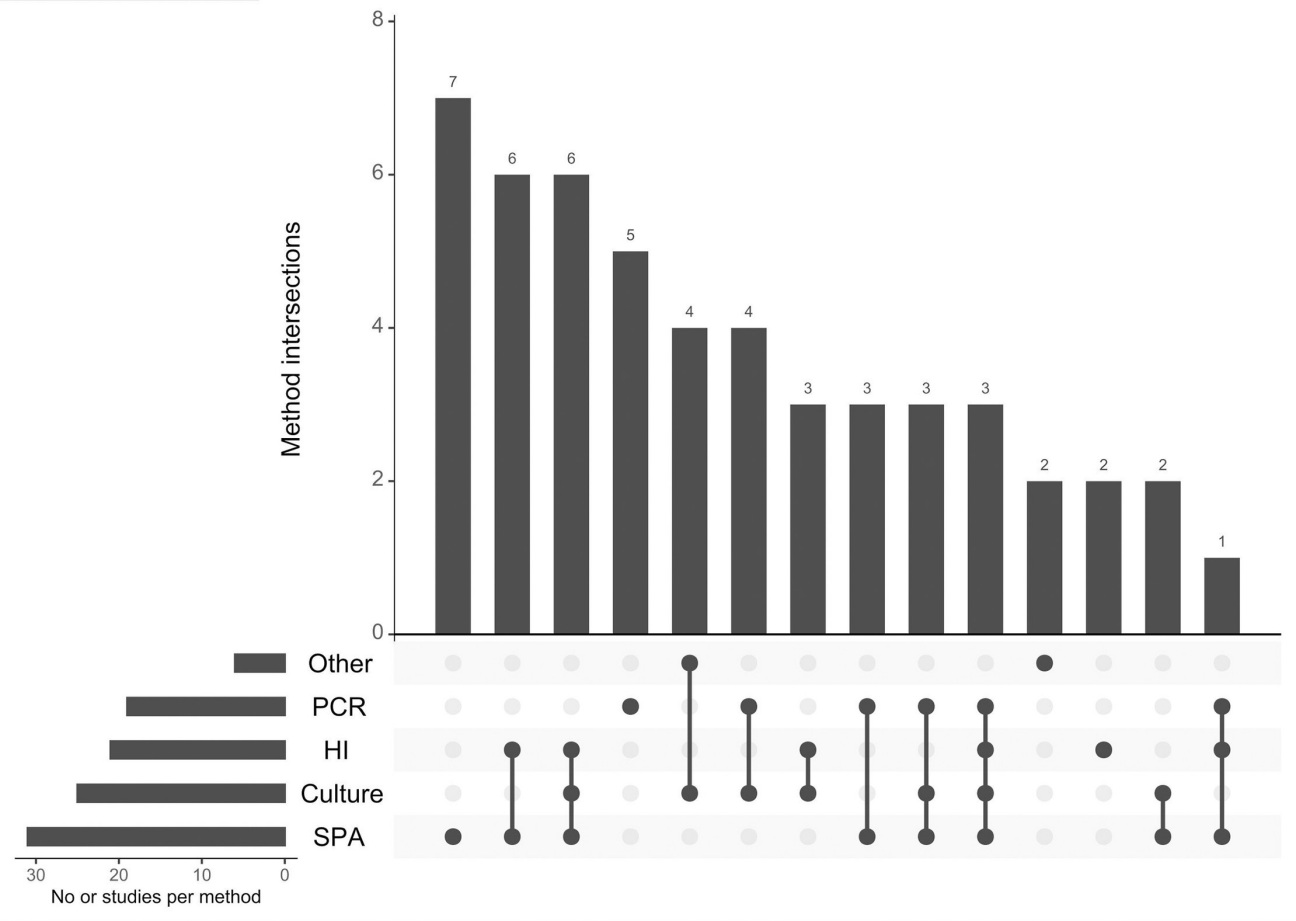
The UpSetR plot showing the frequency of the use of a particular method and their intersections in studies on the occurrence of *Mycoplasma gallisepticum* in wild birds from 1951 to 2018. The horizontal bars with labels at the lower left of the panel represent five methods, with the length of each bar displaying the total number of studies. The dot pattern shows the intersections between methods used and the vertical bars at the top of the plot show the number of the corresponding intersection, ranked by a decreasing number of studies.

We revealed that in 22 studies, the authors used both methods that detect the presence of the pathogen (culture and PCR methods) and anti-MG antibodies. Nineteen studies reported only the result of MG seroprevalence and 11 articles used only methods of pathogen detection. A culture method, PCR and serological tests were used in 5 studies, a culture method and PCR were used in 3 studies, a culture method and serological tests were used in 11 studies, and serological tests and PCR in 6 studies. In 5 articles the authors used only PCR and in 20 articles only serological tests. The most frequent serological test was SPA (31/52 articles), followed by HI (21/52) and ELISA (6/52) (Fig 3).

**Fig 4:**
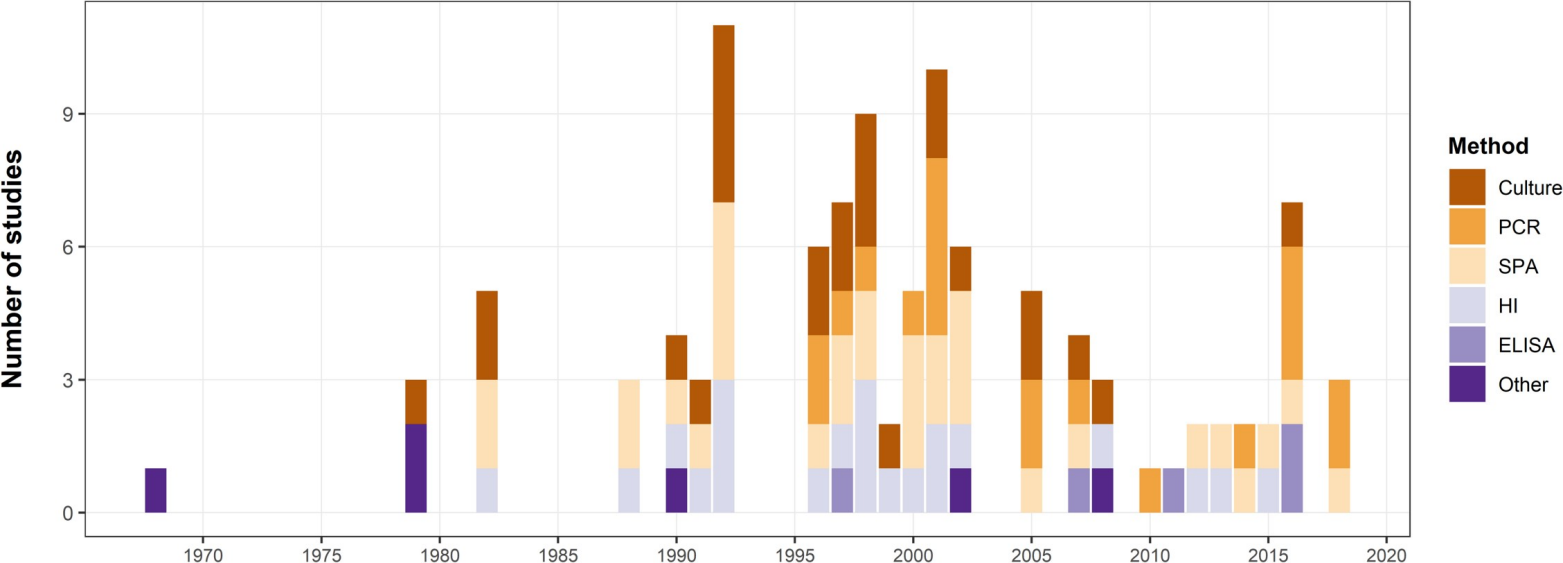
The barplot showing the number of studies by year of publication. Colors represent a particular diagnostic method.

The number of studies using a particular method with the year of publication was shown in Fig 4.

*Mycoplasma gallisepticum* was cultured from 11 passeriform, 1 falconiform and 1 galliform species. The genetic material of MG was found in 36 species belonging to 6 orders. The presence of antibodies against MG was found by SPA in 33 bird species belonging to 16 families and 6 orders and by HI in 10 species from 4 orders including *Accipitriformes*, *Galliformes*, *Falconiformes and Passeriformes*. By ELISA test, the presence of immunoglobulins against MG was found in 7 species belonging to 5 orders. By other serological methods, the presence of anti-MG antibodies was confirmed in 2 species. All information regarding MG-positive results in different species of wild birds and different countries is summarized in Table 1.

**Table 1:** The number of MG-positive birds (number of positive birds/number of birds tested) by taxonomy, country and method of detection.

Order	Family	Species common name (scientific name)	Culture	PCR	SPA	HI	ELISA	Other	Country	No. of studies	References
***Accipitriformes***	*Cathartidae*	black vulture (*Coragyps atratus*)			1/9	0/1			Brazil	1	[47]
***Accipitriformes***	*Cathartidae*	California condor (*Gymnogyps californianus*)			17/120	17/17			USA	1	[48]
***Anseriformes***	*Anatidae*	mallard (*Anas platyrhynchos*)		1/57			1/57		Belgium	1	[49]
***Columbiformes***	*Columbidae*	common wood pigeon (*Columba palumbus*)		1/80			1/80		Belgium	1	[49]
***Columbiformes***	*Columbidae*	mourning dove (*Zenaida macroura*)	0/54	3/54	0/54				USA	1	[18]
***Falconiformes***	*Falconidae*	peregrine falcon (*Falco peregrinus*)	2/2					2/2	Spain	1	[50]
***Falconiformes***	*Falconidae*	prairie falcon (*Falco mexicanus*)				1/34			USA	1	[51]
***Galliformes***	*Phasianidae*	wild turkey (*Meleagris gallopavo*)	4/912	0/1	319/3431	36/1718	0/86	12/250	USA	13	[8,52, 53–60,61–63]
***Galliformes***	*Phasianidae*	lesser prairie-chicken (*Tympanuchius pallidicinctus*)			8/162				USA	1	[64]
***Passeriformes***	*Corvidae*	Eurasian magpie (*Pica pica*)		1/10			1/10		Belgium	1	[49]
***Passeriformes***	*Fringillidae*	evening grosbeak (*Coccothraustes vespertinus*)	1/2	1/1					Canada	1	[65]
***Passeriformes***	*Fringillidae*	pine grosbeak (*Pinicola enucleator*)	0/3	3/3					Canada	1	[65]
***Passeriformes***	*Passeridae*	Eurasian tree sparrow (*Passer montanus*)	6/94					4/94	Japan	1	[10]
***Passeriformes***	*Corvidae*	house crow (*Corvus splendens*)	44/148	4/94	0/45		27/45		Malaysia	1	[66]
***Passeriformes***	*Corvidae*	rook (*Corvus frugilegus*)	0/13	4/13					UK	1	[67]
***Passeriformes***	*Bombycillidae*	cedar waxwing (*Bombycilla garrulus*)		1/10	0/10				USA	1	[17]
***Passeriformes***	*Cardinalidae*	northern cardinal (*Cardinalis cardinalis*)	0/55	1/83	14/83	0/6			USA	3	[17,18,68]
***Passeriformes***	*Corvidae*	western scrub-jay (*Aphelocoma californica*)	1/1	2/2					USA	2	[16,20]
***Passeriformes***	*Corvidae*	American crow (*Corvus brachyrhynchos*)	1/2	2/2					USA	1	[20]
***Passeriformes***	*Corvidae*	blue jay (*Cyanocitta cristata*)	2/6	2/5	1/3				USA	3	[13,18,20]
***Passeriformes***	*Fringillidae*	pine siskin (*Carduelis pinus*)		2/154	3/154				USA	1	[17]
***Passeriformes***	*Fringillidae*	evening grosbeak (*Coccothraustes vespertinus*)	2/2	2/2					USA	1	[20]
***Passeriformes***	*Fringillidae*	house finch (*Haemorhous mexicanus*)	299/524	406/1384	359/1356	48/313	14/52		USA	11	[13,14,16,17,19,20,27,68–70,71]
***Passeriformes***	*Fringillidae*	purple finch (*Haemorhous purpureus*)	3/3	6/37	3/52				USA	4	[16,17,19,20]
***Passeriformes***	*Fringillidae*	lesser goldfinch (*Spinus psaltria*)	2/2	4/4					USA	2	[16,20]
***Passeriformes***	*Fringillidae*	American goldfinch (*Spinus tristis*)	5/51	26/643	18/590				USA	6	[16–20,69]
***Passeriformes***	*Icteridae*	red-winged blackbird (*Agelaius phoeniceus*)	0/1	3/75	1/75				USA	2	[17,18]
***Passeriformes***	*Icteridae*	brown-headed cowbird (*Molothrus ater*)	0/184	0/21	16/201	1/167			USA	5	[17–19,68,72]
***Passeriformes***	*Icteridae*	common grackle (*Quiscalus quiscula*)	0/143	0/3	87/143	50/136			USA	2	[68,72]
***Passeriformes***	*Icteridae*	eastern meadowlark (*Sturnella magna*)	0/24		4/24	4/24			USA	1	[72]
***Passeriformes***	*Mimidae*	gray catbird (*Dumetella carolinensis*)	0/2	0/47	5/47				USA	2	[17,18]
***Passeriformes***	*Mimidae*	northern mockingbird (*Mimus polyglottos*)	0/12	0/12	4/12	0/1			USA	2	[18,68]
***Passeriformes***	*Mimidae*	brown thrasher (*Toxostoma rufum*)	0/9	0/9	4/9				USA	1	[18]
***Passeriformes***	*Paridae*	tufted titmouse (*Baeolophus bicolor*)	0/45	12/81	41/89	7/25			USA	4	[17–19,68]
***Passeriformes***	*Paridae*	black-capped chickadee (*Poecile atricapillus*)	0/1	1/161	11/160				USA	2	[17,20]
***Passeriformes***	*Paridae*	Carolina chickadee (*Poecile carolinensis*)	0/18	0/18	3/18				USA	2	[18,68]
***Passeriformes***	*Parulidae*	common yellowthroat (*Geothlypis trichas*)		1/13	0/13				USA	1	[17]
***Passeriformes***	*Parulidae*	yellow-rumped warbler (*Setophaga coronata*)	0/1	0/1	1/1	0/1			USA	1	[68]
***Passeriformes***	*Passerellidae*	dark-eyed junco (*Junco hyemalis*)		1/15	0/15				USA	1	[17]
***Passeriformes***	*Passerellidae*	song sparrow (*Melospiza melodia*)		1/121	7/121				USA	1	[17]
***Passeriformes***	*Passerellidae*	American tree sparrow (*Spizella arborea*)		1/46	2/46				USA	1	[17]
***Passeriformes***	*Passerellidae*	chipping sparrow (*Spizella passerina*)	0/21	0/21	5/21				USA	2	[18,68]
***Passeriformes***	*Passerellidae*	white-throated sparrow (*Zonotrichia albicollis*)	0/3	1/24	4/24	0/3			USA	2	[17,68]
***Passeriformes***	*Passerellidae*	white-crowned sparrow (*Zonotrichia leucophrys*)		1/23	1/23				USA	1	[17]
***Passeriformes***	*Passeridae*	house sparrow (*Passer domesticus*)	0/348	1/144	19/459	3/309			USA	3	[17,18,68,72]
***Passeriformes***	*Sturnidae*	common starling (*Sturnus vulgaris*)	0/98	0/1	1/98	1/98			USA	2	[68,72]
***Passeriformes***	*Turdidae*	American robin (*Turdus migratorius*)	0/3	0/22	6/22	0/3			USA	2	[17,68]
***Pelecaniformes***	*Ardeidae*	grey heron (*Ardea cinerea*)		2/4			2/4		Belgium	1	[49]
***Piciformes***	*Picidae*	downy woodpecker (*Picoides pubescens*)		1/36	1/36				USA	1	[17]
***Psittaciformes***	*Psittacidae*	Amazon parrot (*Amazona aestiva*)		31/59					Brazil	1	[73]
***Psittaciformes***	*Psittacidae*	orange-winged parrot (*Amazona amazonica*)		1/2					Brazil	1	[73]
***Psittaciformes***	*Psittacidae*	blue-and-yellow macaw (*Ara ararauna*)		4/5					Brazil	1	[73]
***Psittaciformes***	*Psittacidae*	jandaya parakeet or jandaya conure (*Aratinga jandaya*)		1/2					Brazil	1	[73]
***Psittaciformes***	*Psittacidae*	dusky parrot (*Pionus fuscus*)		2/3					Brazil	1	[73]
***Sphenisciformes***	*Spheniscidae*	African penguin (*Spheniscus demersus*)			19/234		1/189		Namibia and South Africa	1	[74]
***Tinamiformes***	*Tinamidae*	red-winged tinamou (*Rhynchotus rufescens*)			3/40	0/40			Brazil	1	[75]
***Tinamiformes***	*Tinamidae*	solitary tinamou (*Tinamus solitarius*)			1/20	0/20			Brazil	1	[75]

### 3.2. Findings from the meta-analysis of prevalence values

In all of the included individual studies 223 species were tested for the presence of MG. A total of 99 species were tested by a culture method, 104 by PCR, 92 by SPA, 63 by HI, 28 by ELISA and 50 by other methods. MG was detected in 56/223 species of birds representing 11 orders. In the 7 orders *Anseriformes*, *Columbiformes*, *Falconiformes*, *Galliformes*, *Passeriformes*, *Pelecaniformes*, and *Piciformes* the presence of the pathogen was confirmed by culture method or PCR, and anti-MG antibodies were detected. Because different detection techniques were used to confirm the presence of MG in one animal, we decided to perform the meta analyses separately for each method.

#### 3.2.1. Culture method

**Fig 5:**
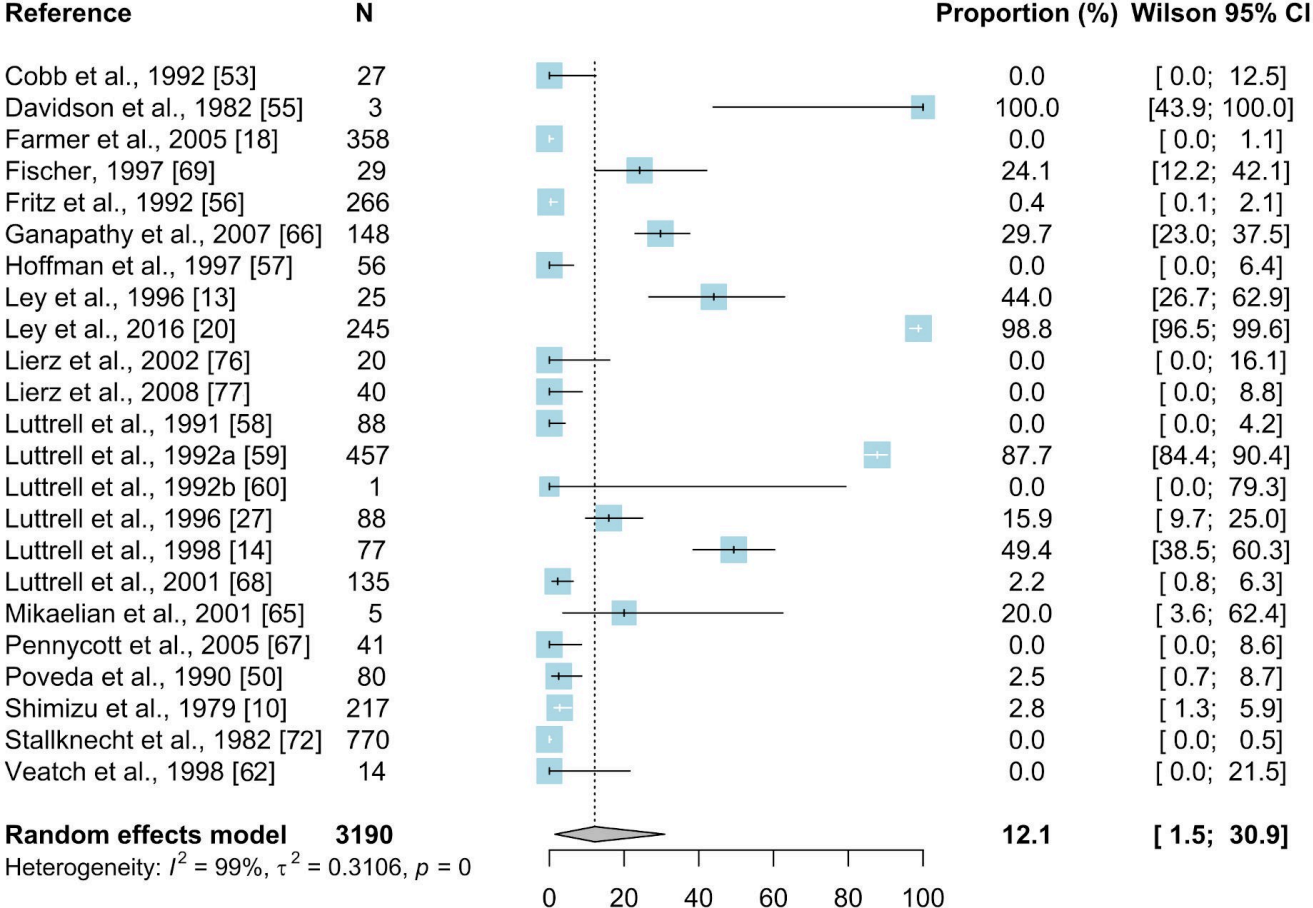
Forest plot of the random effect meta-analysis of MG prevalence (%) calculated based on the results from a culture method. Blue squares represent prevalence of MG of individual studies; the size of each blue square represents the weight of the individual study in calculating average prevalence; horizontal lines represent 95% Wilson confidence intervals (%) of the prevalence from individual studies; the gray diamond and vertical dotted line represent average prevalence calculated using the random-effects model; and I^2^ and τ^2^ statistics describe the heterogeneity of the studies with p-value of the heterogeneity test. [76,77].

For the culture method meta-analysis, 126 prevalence values were extracted from 23 studies and included in the calculation. The mean prevalence of *Mycoplasma gallisepticum* in wild birds was estimated based on a total of 3190 sampled birds and was 12.1% (95% CI: 1.5–30.9) (Fig 5).

Subgroup meta-analyses were performed for geographic region, country, order, species, and captive versus wild birds (S5 Table). Results from culture methods were reported from 6 countries located in Asia, Europe and North America. The highest number of studies focused on *Passeriformes* (N = 1997; 15.8%; 95%CI: 1.2–42.1), and the most frequently sampled species among this order were the house finch (*Haemorhous mexicanus*) (524; 41.2%; 95%CI: 3.6–86.9%) and house sparrow (393; 0%; 95%CI: 0–0.2). The second most frequently reported order was *Galliformes* (N = 953; 7.6%, 95%CI: 0–45.3) with the wild turkey the predominant species (N = 912; 11.8%; 95% CI: 0–58.9). Most of the articles (16/23) reported more frequent occurrence of MG in wild birds (N = 2696; 10.7%; 95%CI: 0.1–35.7) than in captive ones (S5 Table).

#### 3.2.2. Polymerase chain reaction

**Fig 6:**
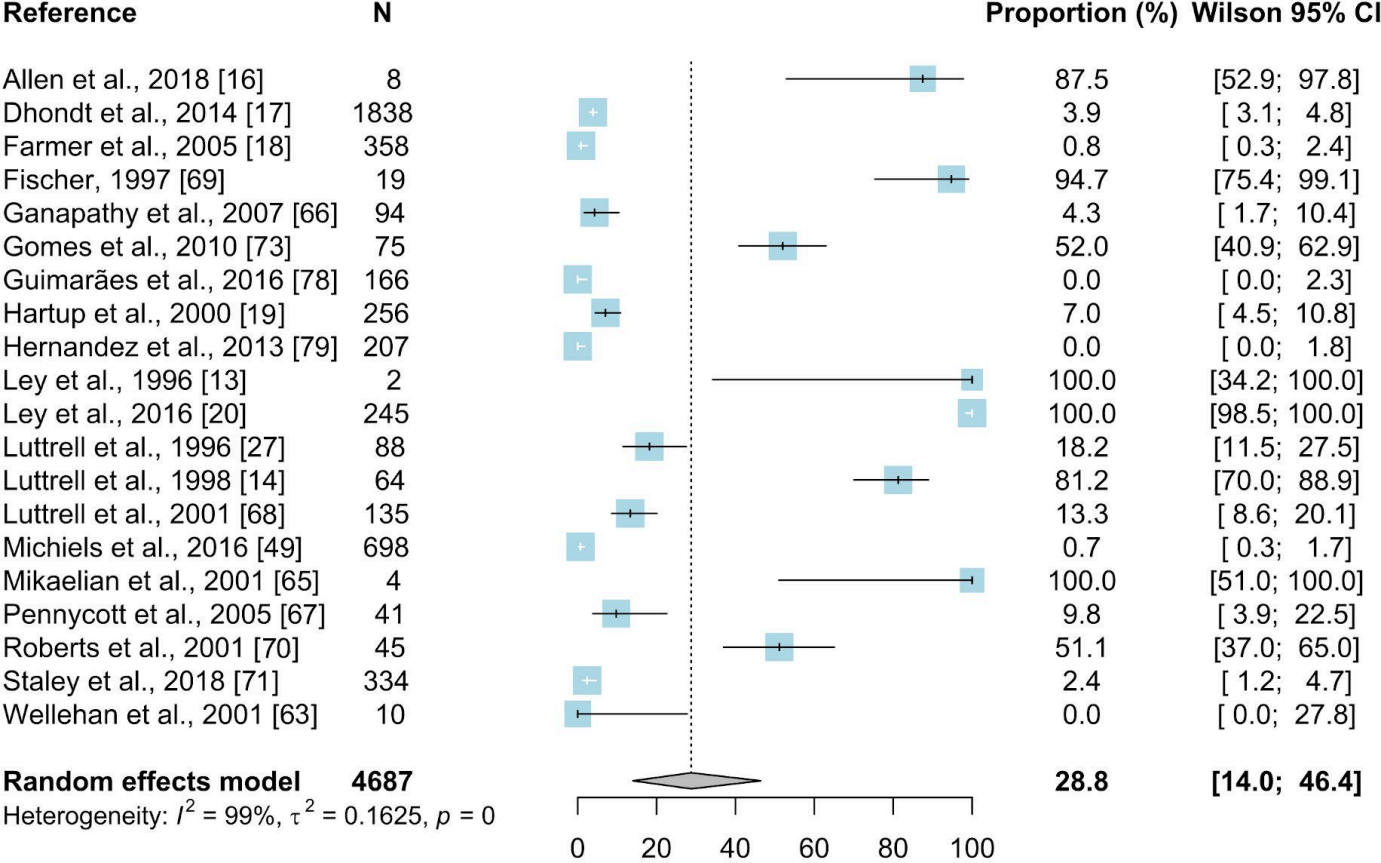
Forest plot of the random effect meta-analysis of MG prevalence (%) calculated based on the results from PCR. Blue squares represent prevalence of MG of individual studies; the size of each blue square represents the weight of the individual study in calculating average prevalence; horizontal lines represent 95% Wilson confidence intervals (%) of the prevalence from individual studies; the gray diamond and vertical dotted line represent average prevalence calculated using the random-effects model; and I^2^ and τ^2^ statistics describe the heterogeneity of the studies with p-value of the heterogeneity test.

PCR was used in 20 studies, and 160 prevalence inputs were extracted from these papers. The resulting mean prevalence of *Mycoplasma gallisepticum* in wild birds was estimated based on a total of 4687 sampled birds and was 28.8% (95% CI: 14.0–46.6) (Fig 6).

Subgroup meta-analyses were performed as mentioned above and the results are presented S6 Table. The results of PCR method for detection of MG in wild birds were reported from 7 countries located in Asia, Europe North and South America. The highest number of studies (19/20) focused on *Passeriformes* (N = 4237; 27.3%; 95%CI: 12.0–46.1), and the most frequently sampled species within this order were the house finch (N = 1384; 53.5%; 95%CI: 20.8–84.6%), American goldfinch (N = 643; 29.3%; 95% CI: 7.7–57.9), and house sparrow (N = 605; 0.03%; 95%CI: 0–0.3). The second most frequently reported order (4/21 papers) was *Columbiformes* (N = 190; 1.5%; 95%CI: 0.03–5.0). The status of captivity was unknown in the majority of papers (11/20) and the prevalence estimated based on these studies was 26.9% (N = 2454; 95%CI: 12.9–43.8).

#### 3.2.3. Serum plate agglutination test

**Fig 7:**
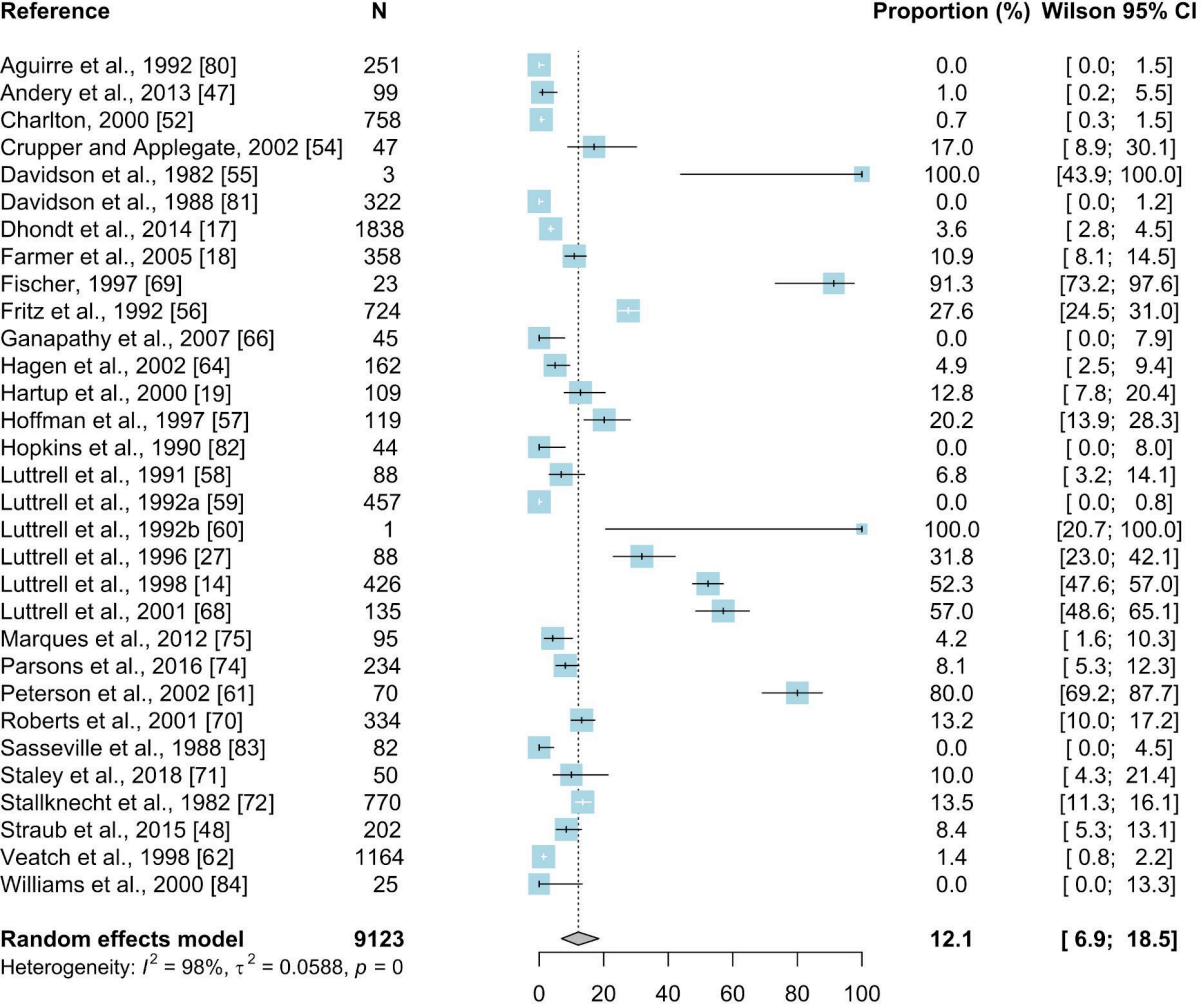
Forest plot of the random effect meta-analysis of MG prevalence (%) calculated based on the results from SPA. Blue squares represent prevalence of MG of individual studies; the size of each blue square represents the weight of the individual study in calculating average prevalence; horizontal lines represent 95% Wilson confidence intervals (%) of the prevalence from individual studies; the gray diamond and vertical dotted line represent average prevalence calculated using the random-effects model; and I^2^ and τ^2^ statistics describe the heterogeneity of the studies with p-value of the heterogeneity test. [78–80,81–83,84].

For the meta-analysis of the SPA method a total of 150 prevalence inputs were extracted from 31 papers. The resulting mean seroprevalence of *Mycoplasma gallisepticum* in wild birds was estimated based on a total of 9123 samples and was 12.1% (95% CI: 6.9–18.5) (Fig 7).

The results of subgroup meta-analyses are summarized in S7 Table. The results of the SPA method were reported from 5 countries located in Africa, Asia, and North and South America. The highest number of studies (17/31) focused on *Galliformes* (N = 4089; 7.8%; 95%CI: 2.3–16.1), and the most frequently sampled species within this order was the wild turkey (N = 3883; 10.7%; 95%CI: 3.3–21.6%). The second most frequently reported order (12/31 papers) was *Passeriformes* (N = 4085; 19.9%; 95%CI: 9.5–32.9) with the house finch (N = 1356, 28.4%; 95% CI: 11.9–48.7), American goldfinch (N = 590; 21.6%; 95% CI 2.6–52.0), and brown-headed cowbird (*Molothrus ater*) (N = 205; 20%; 95% CI: 3.1–46.6) as the most frequently sampled species within the order. The majority of papers (24/31) concerned wild birds (N = 5673; 11.3%; 95% CI: 5.8–18.3) rather than captive ones.

#### 3.2.4. Hemagglutination inhibition test

The analysis of studies which used the HI assay showed that the authors used different titer cutoffs for considering a sample positive. A dilution of ≥1:80 was used as a cutoff level in 6/21 studies, ≥1:40 in 6/21 studies, and ≥1:20 in 1/21 studies. The goal of some authors was only to determine if birds had had contact with the pathogen and in this case titers ≥1:10 were considered MG-positive (2/21 articles). A total of 6 out of 21 studies did not report the cutoff level at all (Table 2). In our summary, we have incorporated all positive results considering the same cutoff titers as stated in the original papers.

**Table 2:** Different cutoff titers used for MG diagnostics by HI assay.

Cutoff titer	Number of studies	References
≥10	2	[61,72]
≥20	1	[52]
≥40	6	[14,27,58,68,79,81]
≥80	6	[48,51,53,56,57,82]
not specified	6	[47,59,62,75,85,86]

**Fig 8:**
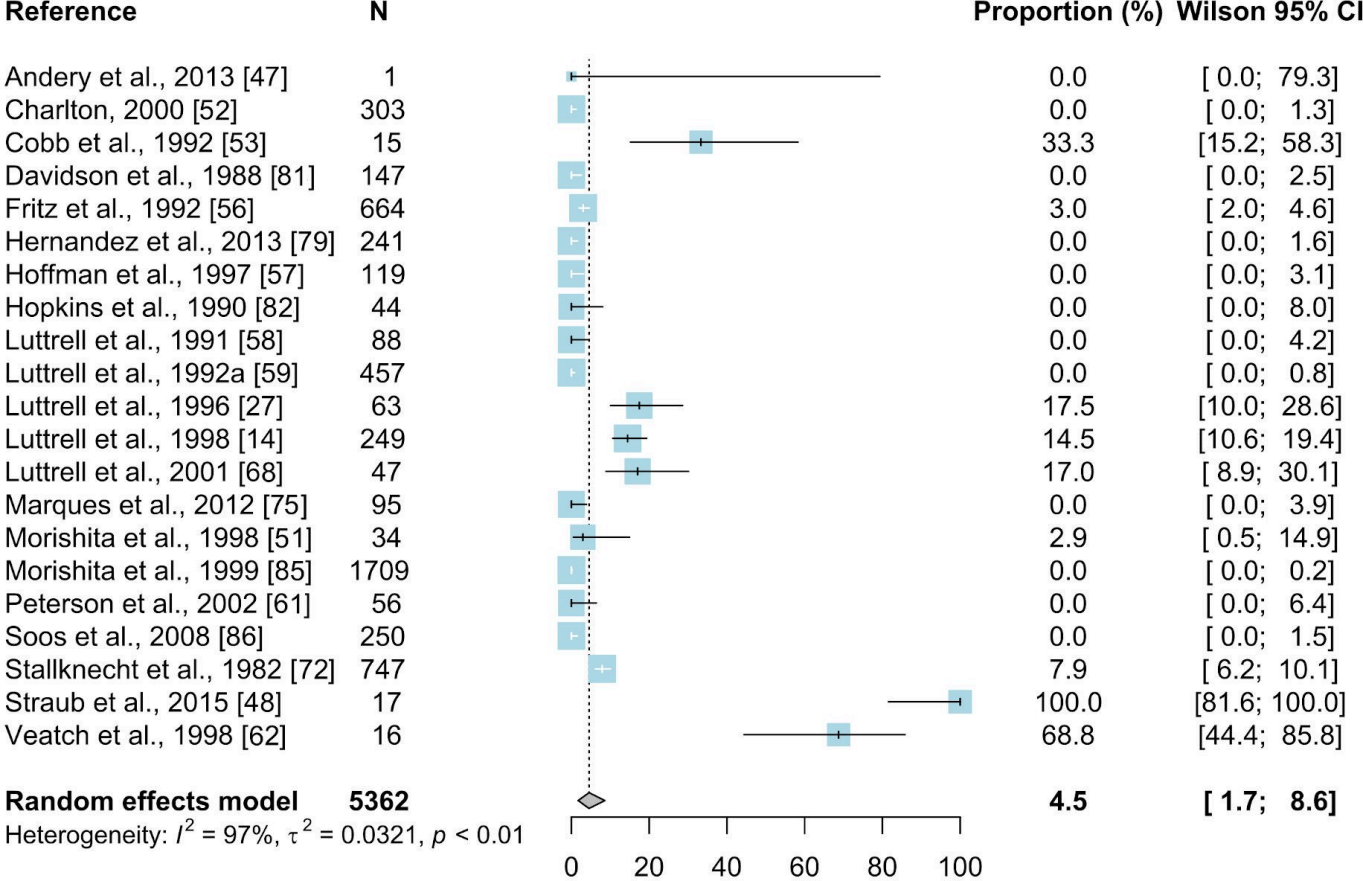
Forest plot of the random effect meta-analysis of MG prevalence (%) calculated based on the results from HI. Blue squares represent prevalence of MG of individual studies; the size of each blue square represents the weight of the individual study in calculating average prevalence; horizontal lines represent 95% Wilson confidence intervals (%) of the prevalence from individual studies; the gray diamond and vertical dotted line represent average prevalence calculated using the random-effects model; and I^2^ and τ^2^ statistics describe the heterogeneity of the studies with p-value of the heterogeneity test.

The hemagglutination inhibition test was used in a total of 21 studies giving 81 prevalence inputs that were used for meta-analysis. The resulting mean seroprevalence of MG in wild birds was estimated based on a total of 5362 samples and was 4.5% (95% CI: 1.7–8.6) (Fig 8). The results of subgroup meta-analyses for HI are summarized in S8 Table. The seroprevalence of MG by HI test was described in articles from North America (USA and Costa Rica, 18/21 papers) and South America (Brazil and Galápagos Islands, 3/21 papers). The highest number of studies (17/21) was from the USA, where the estimated prevalence was 6.6% (95% CI: 2.7–12.2).

No seroprevalence by HI was reported from the rest of world. HI test result reports were reported in 11 out of 21 papers in *Galliformes* (N = 1916; 1.4%; 95%CI: 0.08–4.4) with the wild turkey as the most frequently sampled species again (N = 1909; 1.6%; 95%CI: 0.1–4.8%). The second order was *Passeriformes* (7/21 papers) with estimated prevalence of 4.5% (N = 3263; 95%CI: 0.5–12.3) and here the common starling (*Sturnus vulgaris*) (N = 966, 0.1%,; 95% CI: 0–1.3), house finch (N = 729; 12.7%; 95% CI 0.4–38.0) and house sparrow (N = 682; 0.2%; 95% CI: 0–2.1) were the most frequently sampled species within the order. Once again the majority of papers (17/21) concerned wild birds (N = 4652; 2.3%; 95% CI: 0.5–5.3) rather than captive ones.

#### 3.2.5. Enzyme-linked immunosorbent assay

**Fig 9:**
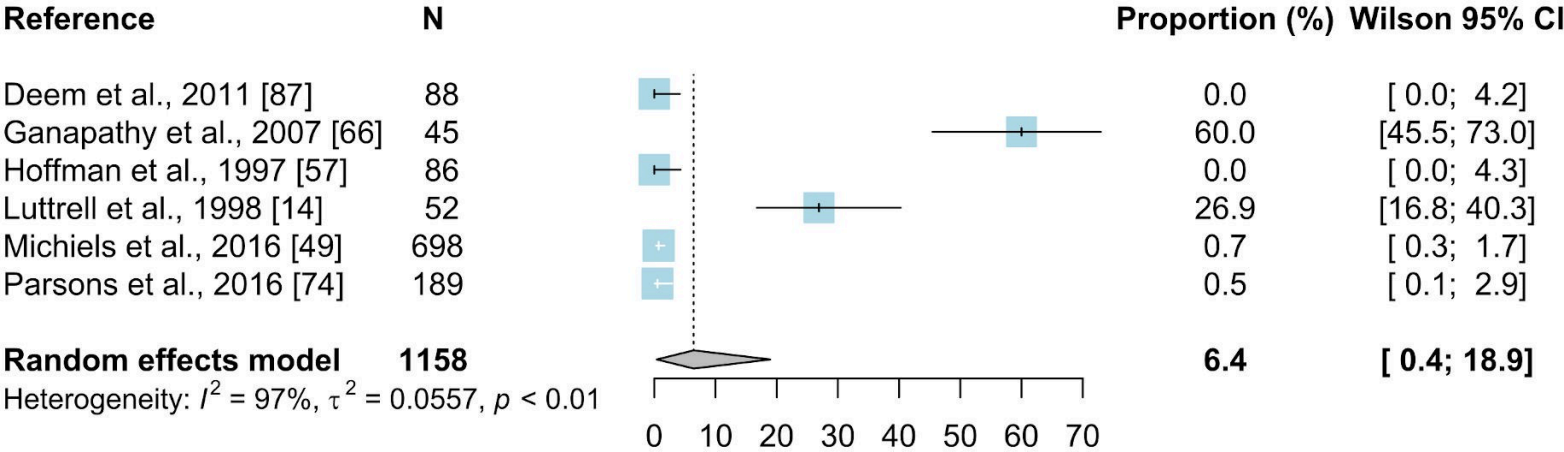
Forest plot of the random effect meta-analysis of MG prevalence (%) calculated based on the results from ELISA. Blue squares represent prevalence of MG of individual studies; the size of each blue square represents the weight of the individual study in calculating average prevalence; horizontal lines represent 95% Wilson confidence intervals (%) of the prevalence from individual studies; the gray diamond and vertical dotted line represent average prevalence calculated using the random-effects model; and I^2^ and τ^2^ statistics describe the heterogeneity of the studies with p-value of the heterogeneity test. [87].

The ELISA was used in six papers that included 28 prevalence inputs. The estimated mean prevalence based on 1158 birds sampled was 6.4% (95%CI: 0.4–18.9) (Fig 9). The results of the subgroup meta-analysis are summarized in S9 Table. Five countries representing five regions were the locations where the reported studies were carried out. *Passeriformes* were the most frequently studied order with estimated prevalence of 12.7% (N = 646; 95% CI: 0.01–43.7) and the house sparrow was the most frequently sampled species (N = 401; 0%; 95% CI: 0–0.2), followed by the house finch (N = 52; 26.9%; 95% CI: 15.9–39.7) and house crow (*Corvus splendens*) (N = 45; 60%; 95% CI: 45.5–73.7). Samples obtained from wild birds were reported in four articles, which resulted in estimated prevalence of 0.4% (N = 1061; 95% CI: 0.04–1.0).

#### 3.2.6. Other methods

**Fig 10:**
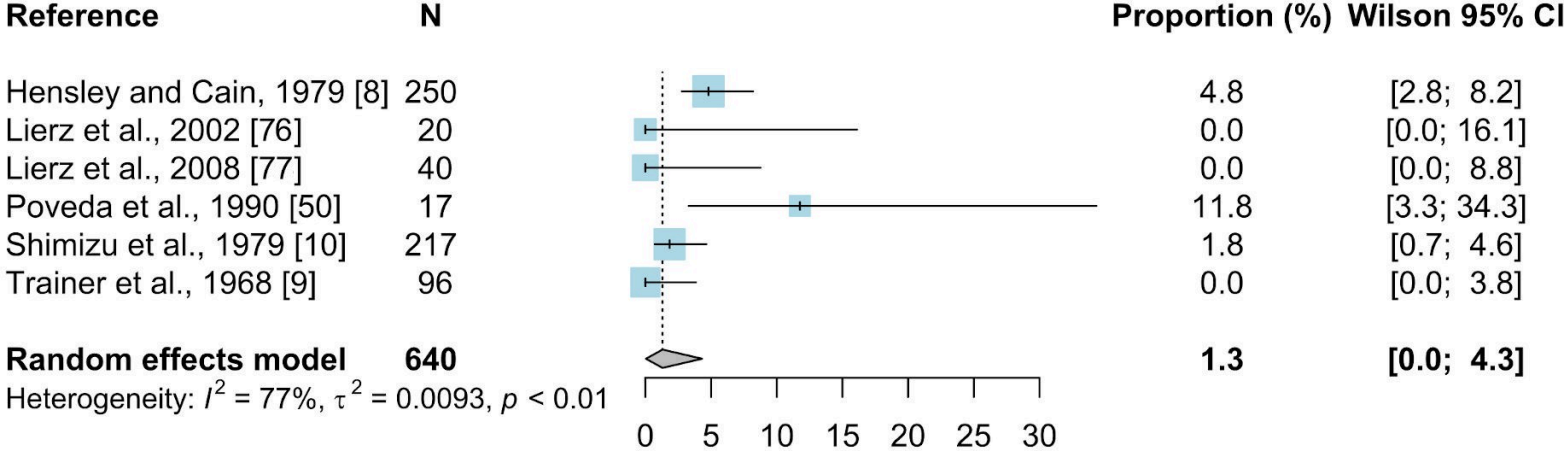
Forest plot of the random effect meta-analysis of MG prevalence (%) calculated based on the results from other methods. Blue squares represent prevalence of MG of individual studies; the size of each blue square represents the weight of the individual study in calculating average prevalence; horizontal lines represent 95% Wilson confidence intervals (%) of the prevalence from individual studies; the gray diamond and vertical dotted line represent average prevalence calculated using the random-effects model; and I^2^ and τ^2^ statistics describe the heterogeneity of the studies with p-value of the heterogeneity test.

The MG was tested by other methods such as the growth inhibition test (GI) and tube agglutination assay (TA) in four studies that were from Germany, Japan, Spain, the United Arab Emirates, and the USA. From these articles a total of 54 prevalence inputs were extracted for meta-analyses. The overall prevalence estimated was 1.3% (N = 640; 95%CI: 0.0–4.3) (Fig 10). Subgroup analysis by order revealed that *Galliformes* (N = 380; 0.7%; 95% CI: 0–6.3) was the most studied one with the wild turkey as the predominant species having mean estimated prevalence of 1.3% (N = 346; 95% CI: 0–10.5). The presence of MG was also detected in the Eurasian tree sparrow (N = 94; 4.3%; 95% CI: 1.1–9.2) and peregrine falcon (*Falco peregrinus*). Five studies described the occurrence of MG in wild birds at 0.9% (N = 575; 95% CI: 0–3.9) and three studies concerned captive birds (N = 65; 1.9%; 95% CI: 0–15.9) (S10 Table).

## 4. Discussion

Many epidemiological aspects of MG infection concerning the pathogen’s carriers, contagiousness, and spread in different geographic areas and among different species of birds are not widely understood. The increase in the number of MG reservoirs could disrupt the eradication of the pathogen in poultry, especially in developing countries in which the appropriate survey and eradication programs have not been introduced and biosecurity requirements are not met [47,78]. Knowing the range of potential hosts of MG and mechanisms of infection spread among species is important from the epidemiological point of view, but systematic epidemiological reviews and meta-analyses on the occurrence of MG in wild birds have not been carried out yet. In our work, we used a comprehensive approach to identify studies on MG detection in wild bird hosts across the world. This study was undertaken to summarize data from over 60 years of research in this field and is a necessary step in identifying data gaps to encourage future research directions. It is not surprising that the majority of studies were from North America, due to the MG epidemic that affects the population of house finches and the constant presence of this pathogen in the wild turkey. The knowledge of MG occurrence in wild birds from the rest of the world is limited to a few papers. Our review included the results of 52 studies focused on testing for MG using different diagnostic methods, including pathogen detection by culture and PCR as well as serological and other assays (Fig 4). The incidences of MG were confirmed in different taxonomically distinct species of wild birds. The MG infection could develop characteristic clinical signs in different species [13,20,55,65,70,88]. MG infection in wild turkeys was confirmed by both culture and serological tests. Although the surveys on the occurrence of MG in this species that were included in this work were conducted between 1968 and 2002, the use of PCR was reported only for one turkey isolate with a negative result [63]. The phylogeny of MG strains isolated from wild turkeys has not yet been established and future studies are needed to determine their origin and compare them with strains isolated from other gallinaceous birds.

MG can spread within members of one order, which could be evidence of a single ancestor [23]. A recent study which summarized the phylogeny of MG showed that one strain of MG could be isolated from three different poultry species [89]. It has now been hypothesized that the adaptation of MG to a new host is not so easy and needs certain favorable factors helping it [90,91]. In 1994, MG was found as a cause of a conjunctivitis epidemic in house finches [13] that spread rapidly across the USA [92]. The outbreak of the disease was caused by the unique strain HFMG, and it led to the substantial decline of the house finch population in the USA at that time [21]. HFMG was reported from different passerines, especially members of the *Fringillidae* family. The disease symptoms in naturally infected birds were found only in the *Fringillidae*. Recent evidence highlights that conjunctivitis caused by the HFMG strain could be also found in the western scrub jay (*Aphelocoma californica*) which belongs to the *Corvidae* family [16]. However, experimental inoculation with HFMG was also able to develop the clinical signs in birds from the *Paridae* family [18]. Although HFMG was found in other *Passeriformes* in the wild, the birds did not show any obvious clinical signs [17]. The previous experimental infection of chickens that were kept with MG-infected house finches showed that clinical disease was not present in the chickens [93] or the symptoms were mild [90]. However, Pflaum et al. [25] demonstrated that different strains of MG originating from house finches (VA1994 and VA2013) induced the disease in experimentally infected chickens, which is clear evidence that MG could be reintroduced from wild hosts to poultry. These results suggested that MG evolved to adapt to the house finch, simultaneously decreasing its virulence for chicken. This study also showed that the virulence of HFMG may be opposite to that found in the original host. The virulence of MG could differ between strains. More recent evidence [94] demonstrates significant differences across strains for factors that are known to cause virulence, including sialidase activity, cytoadherence, and hydrogen peroxide production. There is no obvious inference that a strain that was characterized by mild virulence in the experimental trial could not produce severe symptoms in birds in the field; the synergy of MG and other infectious or environmental factors can result in disease development [95–97]. Also, certain MG strains may not cause visible clinical signs in infected birds [95].

The results for estimated prevalence of MG in wild birds differed between the diagnostic methods. The highest estimated prevalence was obtained based on results of PCR (28.8%, 95% CI: 14–46.4) whereas lower estimated prevalences were found in meta-analyses of studies using other methods (Figs 5–10). Our result suggests that the high prevalence estimated using results from PCR may be due to the higher sensitivity and specificity of this method than other diagnostic methods. The mean estimated MG prevalence obtained using a culture method (12.1%, 95% CI: 1.5–30.9). was similar to that obtained by SPA (12.1%, 95% CI: 6.9–18.5). Culture is considered the performance standard for direct detection of the pathogen, but MG is a fastidious and slow-growing organism and sometimes its isolation can be problematic [53,98,99]. On the other hand, the SPA method may give false-positive results because of its cross-reactivity [100]. However, in some cases SPA may give also false negatives which may result in underestimation of the proportion of MG-positive birds [17,101]. The best solution is to use two different diagnostic methods to maintain a safety margin (Fig 3).

The presence of DNA of MG in the sample or the isolation of the organism by a culture method indicates current infection [99]. Our review revealed that the presence of the pathogen was detected in a total of 36 species by PCR and those species might be considered reservoirs of MG. Additionally, we found 21/106 species of wild birds representing 12 families and 7 orders in which both current and past infection were confirmed by culture method or PCR and serologically.

The majority of bird species that had documented contact with MG were from the USA. Dhondt et al. [17] described the occurrence of MG in 27 of 53 species tested in that country by different methods. In our summary, we used data of 35 studies from the USA, in which the occurrence of MG was detected in 38 out of 53 tested species. The genetic material of MG was found in 23 out of 45 species, while in 14 out of 44 species, MG was confirmed both by PCR or culture and serologically. Current MG infection was reported in 15 species of birds outside the USA. However, two of them (the evening grosbeak (*Coccothraustes vespertinus*) and pine grosbeak (*Pinicola enucleator*)) were found in Canada and the authors suggested that the MG infection was caused by the HFMG strain that caused the epidemic in the USA [65]. The remaining 13 species belonged to distinct taxonomic groups and were indigenous to geographically distant countries. *Mycoplasma gallisepticum* detected in wild birds across the world could represent the past of the recent introduction from backyard poultry, which could be an important reservoir of different MG strains [17]. The phylogenetic analysis of MG strains found in different species of wild birds would be the best evidence of the infection source. The occurrence of MG in wild birds seems only incidental, but due to its small genome (1 Mbp) and high rate of nucleotide substitution [23], MG may adapt to new hosts and there is a possibility of the appearance of MG in new species of birds.

We are aware that our research has some limitations. We included in this work all of the studies concerning the occurrence of MG in a broad spectrum of wild bird species, and most of them reported its occurrence in numerous species. In many cases, sample sizes varied and were less than 10 birds from one species. The second issue was the presence of zero events in some of the included research, which could have a strong influence on the pooled estimated prevalence within subgroups.

Thirdly, a few of the studies lacked information about the clinical signs in tested birds, and in 26% of the studies, we did not find any information as to whether sampled birds were captive or wild. The final number of MG-positive birds was not precisely stated by the authors. Some authors did not describe clearly if samples were tested by one or more serological methods or if different material was taken from different birds. This could lead to misinterpretation because we could not interpret beyond doubt how many birds were finally found MG positive. Another issue was also related to inadequate description of the results. When more than two different samples (e.g. a tracheal swab and an oropharyngeal swab) were taken from one bird, no information was given on which particular sample was found positive. The next issue was the interpretation of the serological results, which was problematical in the lack of agreement between the SPA and HI results we observed in many publications. Another relevant problem was the different values of titer cutoff levels. In cases when samples were tested by two serological methods, the number of samples tested by the second method was different to the number tested by the first one in some of the papers. We are aware that different PCR assays were used in selected research for MG detection, and we were not able to verify their sensitivity and specificity. Taking into account those limitations and the high heterogeneity I^2^ (82% - 99%), the results of our meta-analyses should be considered rather as informative and interpreted with caution. In our work, we wanted to present the whole spectrum of wild bird species in which MG was tested and detected. Thus, we did not focus only on the best estimation of MG prevalence, and we included all of the research that could be helpful to understand which species could be the reservoir or the carrier of MG and how this pathogen could spread between different hosts.

## 5. Conclusions

In this paper, we outlined the current knowledge of the occurrence of MG in wild bird species around the world. Our summary revealed 56 out of 223 tested bird species belonging to different orders and families have documented contact with MG. The majority of MG incidences were reported from the USA due to the disease outbreak in house finches and related species of passerines. In our work, we found 21 species of birds in which past and current infections were confirmed, of which 13 species were from countries distant from the USA. We think that those species could be of interest to other researchers that want to explore the spread of MG and its phylogeny in non-poultry birds. All of the shortcomings that we highlighted may be beneficial for authors of future articles on the current topic, facilitating their better description of their results. We believe that our findings will also emphasize the need for unification of the approach to serological testing for MG and the interpretation of their results.

## Supporting information

S1 Dataset Data extracted from publications included in systematic review and meta-analysis. (XLSX)

S1 Table The PRISMA checklist. (DOCX)

S2 Table Publications included in systematic review and meta-analysis. (DOCX)

S3 Table Number of studies by region and country. (DOCX)

S4 Table Number of studies by order and species of wild birds. (DOCX)

S5 Table Subgroup meta-analysis of the MG prevalence by culture method. (DOCX)

S6 Table Subgroup meta-analysis of the MG prevalence by PCR. (DOCX)

S7 Table Subgroup meta-analysis of the MG prevalence by SPA. (DOCX)

S8 Table Subgroup meta-analysis of the MG prevalence by HI. (DOCX)

S9 Table Subgroup meta-analysis of the MG prevalence by ELISA. (DOCX)

S10 Table Subgroup meta-analysis of the MG prevalence by other methods. (DOCX)

## References

[1] Razin S, Yogev D, Naot Y. Molecular biology and pathogenicity of mycoplasmas. Microbiol Mol Biol Rev. 1998;62: 1094–156. doi: 1092-2172/98 9841667 10.1128/mmbr.62.4.1094-1156.1998 PMC98941

[2] Dhondt AA, Altizer S, Cooch EG, Davis AK, Dobson A, Driscoll MJL, et al Dynamics of a novel pathogen in an avian host: Mycoplasmal conjunctivitis in house finches. Acta Trop. 2005;94: 77–93. 10.1016/j.actatropica.2005.01.009 15777638

[3] Charlton BR, Bermudez AJ, Boulianne M, Eckroade RJ, Jeffrey JS, Newman LJ, et al Avian mycoplasmosis. In: Charlton BR, editor. Avian Disease Manual Pennsylvania: American Association of Avian Pathologists; 1996 pp. 115–125.

[4] Luttrell PM, Fischer JR. Mycoplasmosis. In: Thomas NJ, Hunter BD, Atkinson CT, editors. Infectious Diseases of Wild Birds 1st ed Iowa: Blackwell Publishing; 2007 pp. 317–331.

[5] Saadh MJ, Hasani IW. Isolation and molecular identification of *Mycoplasma gallisepticum* from chicken flocks. J Chem Pharm Res. 2016;8: 721–725.

[6] Nelson JB. Studies on an uncomplicated coryza of the domestic fowl II. The relation of the “bacillary” coryza to that produced by exudate. J Exp Med. 1933;58: 297–304. 10.1084/jem.58.3.297 19870197 PMC2132297

[7] Edward DG f., Kanarek AD. Organisms of the pleuropneumonia group of avian origin: their classification into species. Ann N Y Acad Sci. 1960;79: 696–702. 10.1111/j.1749-6632.1960.tb42744.x 13819382

[8] Hensley TS, Cain JR. Prevalence of certain antibodies to selected disease-causing agents in wild turkeys in Texas. Avian Dis. 1979;23: 62–69. 384989

[9] Trainer DO, Glazener WC, Hanson RP, Nassif BD. Infectious disease exposure in a wild turkey population. Avian Dis. 1968;12: 208–214. 10.2307/1588102 4296150

[10] Shimizu T, Numano K, Uchida K. Isolation and identification of mycoplasmas from various birds: an ecological study. Japanese J Vet Sci. 1979;41: 273–282. 10.1292/jvms1939.41.273 470267

[11] Jessup DA, DaMassa AJ, Lewis R, Jones KR. *Mycoplasma gallisepticum* infection in wild-type turkeys living in close contact with domestic fowl. J Am Vet Med Assoc. 1983;183: 1245–1247. 6643238

[12] Adrian WJ. Investigation of disease as a limiting factor in a wild turkey population. Colorado State University. 1983.

[13] Ley DH, Berkhoff JE, Mclaren JM. Mycoplasma gallisepticum isolated from house finches (*Carpodacus mexicanus*) with conjunctivitis. Avian Dis. 1996;40: 480–483. 10.2307/1592250 8790904

[14] Luttrell MP, Stallknecht DE, Fischer JR, Sewell CT, Kleven SH. Natural *Mycoplasma gallisepticum* infection in a captive flock of house finches. J Wildl Dis. 1998;34: 289–296. 10.7589/0090-3558-34.2.289 9577775

[15] Dhondt AA, Badyaev A V, Dobson AP, Hawley DM, Driscoll MJL, Hochachka WM, et al Dynamics of mycoplasmal conjunctivitis in the native and introduced range of the host. Ecohealth. 2006;3: 95–102. 10.1007/s10393-006-0019-7

[16] Allen CR, Mara A, Tulman ER, Ley DH, Geary SJ. House finch (*Haemorhous mexicanus*)–associated Mycoplasma gallisepticum identified in lesser goldfinch (*Spinus psaltria*) and western scrub jay (*Aphelocoma californica*) using strain-specific quantitative PCR. J Wildl Dis. 2018;54: 180–185. 10.7589/2017-04-079 29053429

[17] Dhondt AA, DeCoste JC, Ley DH, Hochachka WM. Diverse wild bird host range of *Mycoplasma gallisepticum* in Eastern North America. McGraw K, editor. PLoS One. 2014;9: e103553 10.1371/journal.pone.0103553 25061684 PMC4111589

[18] Farmer KL, Hill GE, Roberts SR. Susceptibility of wild songbirds to the house finch strain of *Mycoplasma gallisepticum*. J Wildl Dis. 2005;41: 317–325. 10.7589/0090-3558-41.2.317 16107666

[19] Hartup BK, Kollias G V., Ley DH. Mycoplasmal conjunctivitis in songbirds from New York. J Wildl Dis. 2000;36: 257–264. 10.7589/0090-3558-36.2.257 10813607

[20] Ley DH, Hawley DM, Geary SJ, Dhondt AA. House finch (*Haemorhous mexicanus*) conjunctivitis, and Mycoplasma spp. isolated from North American wild birds, 1994–2015. J Wildl Dis. 2016;52: 669–673. 10.7589/2015-09-244 27285414 PMC4961609

[21] Hochachka WM, Dhondt AA. Density-dependent decline of host abundance resulting from a new infectious disease. Proc Natl Acad Sci. 2000;97: 5303–5306. 10.1073/pnas.080551197 10792031 PMC25823

[22] Nolan PM, Hill GE, Stoehr AM. Sex, size, and plumage redness predict house finch survival in an epidemic. Proc R Soc London Ser B Biol Sci. 1998;265: 961–965. 10.1098/rspb.1998.0384

[23] Delaney NF, Balenger S, Bonneaud C, Marx CJ, Hill GE, Ferguson-Noel N, et al Ultrafast evolution and loss of CRISPRs following a host shift in a novel wildlife pathogen, *Mycoplasma gallisepticum*. PLoS Genet. 2012;8: e1002511 10.1371/journal.pgen.1002511 22346765 PMC3276549

[24] Citti C, Blanchard A. Mycoplasmas and their host: Emerging and re-emerging minimal pathogens. Trends Microbiol. 2013;21: 196–203. 10.1016/j.tim.2013.01.003 23419218

[25] Pflaum K, Tulman ER, Beaudet J, Liao X, Dhondt K V., Dhondt AA, et al Attenuated phenotype of a recent house finch-associated *Mycoplasma gallisepticum* isolate in domestic poultry. Infect Immun. 2017;85: 9–11. 10.1128/IAI.00185-17 28396323 PMC5442625

[26] Garcia M, Jackwood MW, Levisohn S, Kleven SH. Detection of *Mycoplasma gallisepticum*, *M*. *synoviae*, and *M*. *iowae* by multi-species polymerase chain reaction and restriction fragment length polymorphism. Avian Dis. 1995;39: 606 10.2307/1591815 8561747

[27] Luttrell MP, Fischer JR, Stallknecht DE, Kleven SH. Field investigation of *Mycoplasma gallisepticum* infections in house finches (*Carpodacus mexicanus*) from Maryland and Georgia. Avian Dis. 1996;40: 335 10.2307/1592229 8790883

[28] Waites KB, Xiao L, Paralanov V, Viscardi RM, Glass JI. Molecular methods for the detection of mycoplasma and ureaplasma infections in humans: A paper from the 2011 William Beaumont Hospital symposium on molecular pathology. J Mol Diagn. 2012; 14: 437–450. 10.1016/j.jmoldx.2012.06.001 22819362 PMC3427874

[29] Kleven SH. Antibody response to avian mycoplasmas. Am J Vet Res. 1975;36: 563–565. 804836

[30] Kleven SH, Pomeroy BS. Characterization of the antibody response of turkeys to *Mycoplasma meleagridis*. Avian Dis. 1971;15: 291 10.2307/1588699 4932187

[31] Martínez J, Tomas G, Merino S, Arriero E, Moreno J. Detection of serum immunoglobulins in wild birds by direct ELISA: a methodological study to validate the technique in different species using antichicken antibodies. Funct Ecol. 2003;17: 700–706. 10.1046/j.1365-2435.2003.00771.x

[32] Ebel GD, Dupuis AP, Nicholas D, Young D, Maffei J, Kramer LD. Detection by enzyme-linked immunosorbent assay of antibodies to West Nile virus in birds. Emerg Infect Dis. 2002;8: 979–982. 10.3201/eid0809.020152 12194778 PMC2732549

[33] Fassbinder-Orth CA, Wilcoxen TE, Tran T, Boughton RK, Fair JM, Hofmeister EK, et al Immunoglobulin detection in wild birds: effectiveness of three secondary anti-avian IgY antibodies in direct ELISAs in 41 avian species. Methods Ecol Evol. 2016;7: 1174–1181. 10.1111/2041-210X.12583 27800150 PMC5084450

[34] Moher D, Liberati A, Tetzlaff J, Altman DG. Preferred reporting items for systematic reviews and meta-analyses: the PRISMA statement. PLoS Med. 2009;6: e1000097 10.1371/journal.pmed.1000097 19621072 PMC2707599

[35] Stroup DF. Meta-analysis of observational studies in epidemiology: a proposal for reporting. JAMA. 2000;283: 2008 10.1001/jama.283.15.2008 10789670

[36] Benskin CMWH, Wilson K, Jones K, Hartley IR. Bacterial pathogens in wild birds: A review of the frequency and effects of infection. Biol Rev. 2009;84: 349–373. 10.1111/j.1469-185X.2008.00076.x 19438430

[37] Faustino CR, Jennelle CS, Connolly V, Davis AK, Swarthout EC, Dhondt AA, et al *Mycoplasma gallisepticum* infection dynamics in a house finch population: seasonal variation in survival, encounter and transmission rate. J Anim Ecol. 2004;73: 651–669. 10.1111/j.0021-8790.2004.00840.x

[38] Hunter JP, Saratzis A, Sutton AJ, Boucher RH, Sayers RD, Bown MJ. In meta-analyses of proportion studies, funnel plots were found to be an inaccurate method of assessing publication bias. J Clin Epidemiol. 2014;67: 897–903. 10.1016/j.jclinepi.2014.03.003 24794697

[39] R Core Team. R: A language and environment for statistical computing. Version 3.6.1. In: Foundation for Statistical Computing, Vienna, Austria [Internet]. 2019 Available: http://www.r-project.org/

[40] Wickham H, Romain F, Lionel H, Müller K. dplyr: A Grammar of Data Manipulation. R package version 0.8.1. [Internet]. 2019. Available: https://cran.r-project.org/package=dplyr

[41] Schwarzer G, Chemaitelly H, Abu‐Raddad LJ, Rücker G. Seriously misleading results using inverse of Freeman‐Tukey double arcsine transformation in meta‐analysis of single proportions. Res Synth Methods. 2019;10: 476–483. 10.1002/jrsm.1348 30945438 PMC6767151

[42] Schwarzer G. meta: An R package for meta-analysis. R News. 2007;7: 40–45. Available: http://link.springer.com/10.1007/978-3-319-21416-0

[43] Newcombe RG. Two-sided confidence intervals for the single proportion: comparison of seven methods. Stat Med. 1998;17: 857–872. 10.1002/(sici)1097-0258(19980430)17:8<857::aid-sim777>3.0.co;2-e 9595616

[44] Wickham H. ggplot2 [Internet]. Media. New York, NY: Springer New York; 2009. 10.1007/978-0-387-98141-3

[45] Fellow I. wordcloud: Word Clouds. R package version 2.6 [Internet]. 2018.

[46] Conway JR, Lex A, Gehlenborg N. UpSetR: an R package for the visualization of intersecting sets and their properties. Bioinformatics. 2017;33: 2938–2940. 10.1093/bioinformatics/btx364 28645171 PMC5870712

[47] Andery D de A, Ferreira Junior F, Araújo A de, Vilela D da R, Marques M, Marin S, et al Health assessment of raptors in triage in Belo Horizonte, MG, Brazil. Rev Bras Ciência Avícola. 2013;15: 247–256. 10.1590/S1516-635X2013000300012

[48] Straub MH, Kelly TR, Rideout BA, Eng C, Wynne J, Braun J, et al Seroepidemiologic survey of potential pathogens in obligate and facultative scavenging avian species in California. PLoS One. 2015;10: e0143018 10.1371/journal.pone.0143018 26606755 PMC4659623

[49] Michiels T, Welby S, Vanrobaeys M, Quinet C, Rouffaer L, Lens L, et al Prevalence of *Mycoplasma gallisepticum* and *Mycoplasma synoviae* in commercial poultry, racing pigeons and wild birds in Belgium. Avian Pathol. 2016;45: 244–252. 10.1080/03079457.2016.1145354 26814376

[50] Poveda JB, Carranza J, Miranda A, Garrido A, Hermoso M, Fernandez A, et al An epizootiological study of avian mycoplasmas in Southern Spain. Avian Pathol. 1990;19: 627–633. 10.1080/03079459008418718 18679976

[51] Morishita TY, McFadzen ME, Mohan R, Aye PP, Brooks DL. Serologic survey of free-living nestling prairie falcons (*Falco mexicanus*) for selected pathogens. J Zoo Wildl Med. 1998;29: 18–20. 10.2307/1592655 9638619

[52] Charlton KG. Antibodies to selected disease agents in translocated wild turkeys in California. J Wildl Dis. 2000;36: 161–164. 10.7589/0090-3558-36.1.161 10682760

[53] Cobb DT, Ley DH, Doerr PD. Isolation of *Mycoplasma gallopavonis* from free-ranging wild turkeys in coastal North Carolina seropositive and culture-negative for *Mycoplasma gallisepticum*. J Wildl Dis. 1992;28: 105–109. 10.7589/0090-3558-28.1.105 1548788

[54] Crupper SS, Applegate RD. Incidence of antibodies to selected bacterial pathogens in wild turkeys (*Meleagris gallopavo*) in Kansas, USA. Vet Rec. 2002;151: 450 10.1136/vr.151.15.450 12408329

[55] Davidson WR, Nettles VF, Couvillion CE, Yoder HW. Infectious sinusitis in wild turkeys. Avian Dis. 1982;26: 402 10.2307/1590112 7103896

[56] Fritz BA, Thomas CB, Yuill TM. Serological and microbial survey of *Mycoplasma gallisepticum* in wild turkeys (*Meleagris gallopavo*) from six western states. J Wildl Dis. 1992;28: 10–20. 10.7589/0090-3558-28.1.10 1548787

[57] Hoffman RW, Page Luttrell M, Davidson WR, Ley DH. Mycoplasmas in wild turkeys living in association with domestic fowl. J Wildl Dis. 1997;33: 526–535. 10.7589/0090-3558-33.3.526 9249699

[58] Luttrell MP, Kleven SH, Davidson WR. An investigation of the persistence of *Mycoplasma gallisepticum* in an Eastern population of wild turkeys. J Wildl Dis. 1991;27: 74–80. 10.7589/0090-3558-27.1.74 2023330

[59] Luttrell MP, Eleazer TH, Kleven SH. *Mycoplasma gallopavonis* in Eastern wild turkeys. J Wildl Dis. 1992;28: 288–291. 10.7589/0090-3558-28.2.288 1602583

[60] Luttrell MP, Kleven SH, Mahnke GM. *Mycoplasma synoviae* in a released pen-raised wild turkey. Avian Dis. 1992;36: 169 10.2307/1591734 1567302

[61] Peterson MJ, Aguirre R, Ferro PJ, Jones DA, Lawyer TA, Peterson MN, et al Infectious disease survey of Rio Grande wild turkeys in the Edwards plateau of Texas. J Wildl Dis. 2002;38: 826–833. 10.7589/0090-3558-38.4.826 12528453

[62] Veatch JK, Applegate RD, Osborne SJ. Serologic incidence of some diseases in Kansas wild turkeys. Avian Dis. 1998;42: 393 10.2307/1592492 9645333

[63] Wellehan JFX, Calsamiglia M, Ley DH, Zens MS, Amonsin A, Kapur V. Mycoplasmosis in captive crows and robins from Minnesota. J Wildl Dis. 2001;37: 547–555. 10.7589/0090-3558-37.3.547 11504228

[64] Hagen CA, Crupper SS, Applegate RD, Robel RJ. Prevalence of mycoplasma antibodies in lesser prairie-chicken sera. Avian Dis. 2002;46: 708–12. 10.1637/0005-2086(2002)046[0708:POMAIL]2.0.CO;2 12243537

[65] Mikaelian I, Ley DH, Claveau R, Lemieux M, Bérubé J-P. Mycoplasmosis in evening and pinegrosbeaks with conjunctivitis in Quebec. J Wildl Dis. 2001;37: 826–830. 10.7589/0090-3558-37.4.826 11763749

[66] Ganapathy K, Saleha AA, Jaganathan M, Tan CG, Chong CT, Tang SC, et al Survey of campylobacter, salmonella and mycoplasmas in house crows (*Corvus splendens*) in Malaysia. Vet Rec. 2007;160: 622–624. 10.1136/vr.160.18.622 17483380

[67] Pennycott TW, Dare CM, Yavari CA, Bradbury JM. *Mycoplasma sturni* and *Mycoplasma gallisepticum* in wild birds in Scotland. Vet Rec. 2005;156: 513–515. 10.1136/vr.156.16.513 15833969

[68] Luttrell MP, Stallknecht DE, Kleven SH, Kavanaugh DM, Corn JL, Fischer JR. *Mycoplasma gallisepticum* in house finches (*Carpodacus mexicanus*) and other wild birds associated with poultry production facilities. Avian Dis. 2001;45: 321 10.2307/1592971 11417811

[69] Fischer J. Mycoplasmal conjunctivitis in wild songbirds: the spread of a new contagious disease in a mobile host population. Emerg Infect Dis. 1997;3: 69–72. 10.3201/eid0301.970110 9126448 PMC2627586

[70] Roberts SR, Nolan PM, Lauerman LH, Li L-Q, Hill GE. Characterization of the mycoplasmal conjunctivitis epizootic in a house finch population in the southeastern USA. J Wildl Dis. 2001;37: 82–88. 10.7589/0090-3558-37.1.82 11272508

[71] Staley M, Bonneaud C, McGraw KJ, Vleck CM, Hill GE. Detection of *Mycoplasma gallisepticum* in house finches (*Haemorhous mexicanus*) from Arizona. Avian Dis. 2018;62: 14–17. 10.1637/11610-021317-Reg.1 29620468

[72] Stallknecht DE, Johnson DC, Emory WH, Kleven SH. Wildlife surveillance during a *Mycoplasma gallisepticum* epornitic in domestic turkeys. Avian Dis. 1982;26: 883–890. 7159324

[73] Gomes AM, Costa LL, Vilela DAR, Marques MVR, Carvalhaes AG, Marin SY, et al Detection of *Mycoplasma gallisepticum* in dead captive psittacines in Belo Horizonte, Brazil. Brazilian J Poult Sci. 2010;12: 75–78.

[74] Parsons NJ, Gous TA, Schaefer AM, Vanstreels RET. Health evaluation of African penguins (Spheniscus demersus) in southern Africa. Onderstepoort J Vet Res. 2016;83: 1–13. 10.4102/ojvr.v83i1.1147 27796116 PMC6238701

[75] Marques MVR, Junior FCF, de Assis Andery D, Fernandes AA, de Araújo AV, de Resende JS, et al Health assessment of captive tinamids (*Aves, Tinamiformes*) in Brazil. J Zoo Wildl Med. 2012;43: 539–548. 10.1638/2011-0262R1.1 23082518

[76] Lierz M, Schmidt R, Runge M. Mycoplasma species isolated from falcons in the Middle East. Vet Rec. 2002;151: 92–93. 10.1136/vr.151.3.92 12164228

[77] Lierz M, Hangen N, Hernandez-Divers J, Hafez HM. Occurrence of mycoplasmas in semen samples of birds of prey. Avian Pathol. 2008;37: 495–497. 10.1080/03079450802356961 18798023

[78] Guimarães M, Hurtado R, Bello C, Vanstreels R, Ferreira A. Surveillance for Newcastle disease virus, Avian influenza virus and *Mycoplasma gallisepticum* in wild birds near commercial poultry farms surrounded by Atlantic Rainforest remnants, Southeastern Brazil. Rev Bras Ciência Avícola. 2016;18: 387–394. 10.1590/1806-9061-2015-0164

[79] Hernandez SM, Peters VE, Weygandt PL, Jimenez C, Villegas P, O’Connor B, et al Do shade-grown coffee plantations pose a disease risk for wild birds? Ecohealth. 2013;10: 145–158. 10.1007/s10393-013-0837-3 23636482

[80] Aguirre AA, McLean RG, Cook RS, Quan TJ. Serologic survey for selected arboviruses and other potential pathogens in wildlife from Mexico. J Wildl Dis. 1992;28: 435–442. 10.7589/0090-3558-28.3.435 1512876

[81] Davidson WR, Yoder HW, Brugh M, Nettles VF. Serological monitoring of eastern wild turkeys for antibodies to *Mycoplasma* spp. and avian influenza viruses. J Wildl Dis. 1988;24: 348–51. 10.7589/0090-3558-24.2.348 3373642

[82] Hopkins BA, Skeeles JK, Houghten GE, Slagle D, Gardner K. A survey of infectious diseases in wild turkeys (*Meleagridis gallopavo silvestris*) from Arkansas. J Wildl Dis. 1990;26: 468–472. 10.7589/0090-3558-26.4.468 2250323

[83] Sasseville VG, Miller B, Nielsen SW. A pathologic study of wild turkeys in Connecticut. Cornell Vet. 1988;78: 353–64. 3168472

[84] Williams CK, Davidson WR, Lutz RS, Applegate RD. Health status of Northern bobwhite quail (*Colinus virginianus*) in Eastern Kansas. Avian Dis. 2000;44: 953 10.2307/1593071 11195653

[85] Morishita TY, Aye PP, Ley EC, Harr BS. Survey of pathogens and blood parasites in free-living passerines. Avian Dis. 1999;43: 549 10.2307/1592655 10494426

[86] Soos C, Padilla L, Iglesias A, Gottdenker N, Bedon MC, Rios A, et al Comparison of pathogens in broiler and backyard chickens on the Galápagos Islands: Implications for transmission to wildlife. Auk. 2008;125: 445–455. 10.1525/auk.2008.06235

[87] Deem SL, Parker PG, Cruz MB, Merkel J, Hoeck PEA. Comparison of blood values and health status of Floreana Mockingbirds (Mimus trifasciatus) on the islands of Champion and Gardner-by-Floreana, Galápagos Islands. J Wildl Dis. 2011;47: 94–106. 10.7589/0090-3558-47.1.94 21270000

[88] Hartup BK, Kollias G V. Field Investigation of *Mycoplasma gallisepticum* infections in house finch (*Carpodacus mexicanus*) eggs and nestlings. Avian Dis. 1999;43: 572 10.2307/1592658 10494429

[89] Bekő K, Kreizinger Z, Sulyok KM, Kovács ÁB, Grózner D, Catania S, et al Genotyping *Mycoplasma gallisepticum* by multilocus sequence typing. Vet Microbiol. 2019;231: 191–196. 10.1016/j.vetmic.2019.03.016 30955809

[90] Bonneaud C, Weinert LA, Kuijper B. Understanding the emergence of bacterial pathogens in novel hosts. Philos Trans R Soc B Biol Sci. 2019;374 10.1098/rstb.2018.0328 31401968 PMC6711297

[91] Staley M, Hill GE, Josefson CC, Armbruster JW, Bonneaud C. Bacterial pathogen emergence requires more than direct contact with a novel passerine host. Infect Immun. 2018;86: 1–9. 10.1128/IAI.00863-17 29311238 PMC5820954

[92] Dhondt AA, Tessaglia DL, Slothower RL. Epidemic mycoplasmal conjunctivitis in house finches from eastern North America. J Wildl Dis. 1998;34: 265–280. 10.7589/0090-3558-34.2.265 9577773

[93] Stallknecht DE, Luttrell MP, Fischer JR, Kleven SH. Potential for transmission of the finch strain of *Mycoplasma gallisepticum* between house finches and chickens. Avian Dis. 1998;42: 352 10.2307/1592485 9645326

[94] Perez K, Mullen N, Canter JA, Ley DH, May M. Phenotypic diversity in an emerging mycoplasmal disease. Microb Pathog. 2020;138: 103798 10.1016/j.micpath.2019.103798 31639466

[95] Kleven SH. Mycoplasmas in the etiology of multifactorial respiratory disease. Poult Sci. 1998;77: 1146–1149. 10.1093/ps/77.8.1146 9706080

[96] Sanei B, Barnes HJ, Vaillancourt JP, Ley DH. Experimental infection of chickens and turkeys with *Mycoplasma gallisepticum* reference strain S6 and North Carolina field isolate RAPD type B. Avian Dis Dig. 2007;2: e16–e16. 10.1637/1933-5334(2007)2[e16:eiocat]2.0.co;2 17461274

[97] Stipkovits L, Kempf I. Mycoplasmoses in poultry. Rev Sci Tech l’OIE. 1996;15: 1495–1525. 10.20506/rst.15.4.986 9190023

[98] García M, Ikuta N, Levisohn S, Kleven SH. Evaluation and comparison of various PCR methods for detection of *Mycoplasma gallisepticum* infection in chickens. Avian Dis. 2005;49: 125–132. 10.1637/7261-0812204R1 15839425

[99] Levisohn S, Kleven SH. Avian mycoplasmosis (*Mycoplasma gallisepticum*). Rev Sci Tech l’OIE. 2000;19: 425–442. 10.20506/rst.19.2.1232 10935272

[100] Feberwee A, Mekkes DR, de Wit JJ, Hartman EG, Pijpers A. Comparison of culture, PCR, and different serologic tests for detection of *Mycoplasma gallisepticum* and *Mycoplasma synoviae* infections. Avian Dis. 2005;49: 260–268. 10.1637/7274-090804R 16094832

[101] Dhondt AA, Dhondt K V., Hochachka WM. Response of black-capped chickadees to house finch *Mycoplasma gallisepticum*. PLoS One. 2015;10: 1–9. 10.1371/journal.pone.0124820 25880849 PMC4400008

