# Supplementary material for: Occurrence of Mycoplasma gallisepticum in wild birds: A systematic review and meta-analysis
Source: PLoS One. 2020 Apr 16;15(4):e0231545. doi: 10.1371/journal.pone.0231545 (PMC7162529; doi:10.1371/journal.pone.0231545)
Supplement: S2 Table — (DOCX) [file pone.0231545.s003.docx]

S2 Table. List of publications included in systematic review and meta-analysis in alphabetical order.

| **Reference** | **Ref. No.** |
| --- | --- |
| Aguirre AA, McLean RG, Cook RS, Quan TJ. Serologic survey for selected arboviruses and other potential pathogens in wildlife from Mexico. J Wildl Dis. 1992;28: 435–442. doi:10.7589/0090-3558-28.3.435 | [77] |
| Allen CR, Mara A, Tulman ER, Ley DH, Geary SJ. House finch (*Haemorhous mexicanus*)–associated *Mycoplasma gallisepticum* identified in lesser goldfinch (*Spinus psaltria*) and western scrub jay (*Aphelocoma californica*) using strain-specific quantitative PCR. J Wildl Dis. 2018;54: 180–185. doi:10.7589/2017-04-079 | [16] |
| Andery D de A, Ferreira Junior F, Araújo A de, Vilela D da R, Marques M, Marin S, et al. Health assessment of raptors in triage in Belo Horizonte, MG, Brazil. Rev Bras Ciência Avícola. 2013;15: 247–256. doi:10.1590/S1516-635X2013000300012 | [44] |
| Charlton KG. Antibodies to Selected Disease Agents in Translocated Wild Turkeys in California. J Wildl Dis. 2000;36: 161–164. doi:10.7589/0090-3558-36.1.161 | [49] |
| Cobb DT, Ley DH, Doerr PD. Isolation of *Mycoplasma gallopavonis* from Free-ranging Wild Turkeys in Coastal North Carolina Seropositive and Culture-negative for *Mycoplasma gallisepticum*. J Wildl Dis. 1992;28: 105–109. doi:10.7589/0090-3558-28.1.105 | [53] |
| Crupper SS, Applegate RD. Incidence of antibodies to selected bacterial pathogens in wild turkeys (*Meleagris gallopavo*) in Kansas, USA. Vet Rec. 2002;151: 450. doi:10.1136/vr.151.15.450 | [54] |
| Davidson WR, Nettles VF, Couvillion CE, Yoder HW. Infectious Sinusitis in Wild Turkeys. Avian Dis. 1982;26: 402. doi:10.2307/1590112 | [55] |
| Davidson WR, Yoder HW, Brugh M, Nettles VF. Serological monitoring of eastern wild turkeys for antibodies to *Mycoplasma* spp. and avian influenza viruses. J Wildl Dis. 1988;24: 348–51. doi:10.7589/0090-3558-24.2.348 | [78] |
| Deem SL, Parker PG, Cruz MB, Merkel J, Hoeck PEA. Comparison of blood values and health status of Floreana Mockingbirds (*Mimus trifasciatus*) on the islands of Champion and Gardner-by-Floreana, Galápagos Islands. J Wildl Dis. 2011;47: 94–106. doi:10.7589/0090-3558-47.1.94 | [84] |
| Dhondt AA, DeCoste JC, Ley DH, Hochachka WM. Diverse wild bird host range of *Mycoplasma gallisepticum* in Eastern North America. McGraw K, editor. PLoS One. 2014;9: e103553. doi:10.1371/journal.pone.0103553 | [17] |
| Farmer KL, Hill GE, Roberts SR. Susceptibility of wild songbirds to the house finch strain of *Mycoplasma gallisepticum*. J Wildl Dis. 2005;41: 317–325. doi:10.7589/0090-3558-41.2.317 | [18] |
| Fischer J. Mycoplasmal Conjunctivitis in Wild Songbirds: The Spread of a New Contagious Disease in a Mobile Host Population. Emerg Infect Dis. 1997;3: 69–72. doi:10.3201/eid0301.970110 | [67] |
| Fritz BA, Thomas CB, Yuill TM. Serological and microbial survey of *Mycoplasma gallisepticum* in wild turkeys (*Meleagris gallopavo*) from six western states. J Wildl Dis. 1992;28: 10–20. doi:10.7589/0090-3558-28.1.10 | [56] |
| Ganapathy K, Saleha AA, Jaganathan M, Tan CG, Chong CT, Tang SC, et al. Survey of campylobacter, salmonella and mycoplasmas in house crows (*Corvus splendens*) in Malaysia. Vet Rec. 2007;160: 622–624. doi:10.1136/vr.160.18.622 | [63] |
| Gomes AM, Costa LL, Vilela DAR, Marques MVR, Carvalhaes AG, Marin SY, et al. Detection *of Mycoplasma gallisepticum* in dead captive psittacines in Belo Horizonte, Brazil. Brazilian J Poult Sci. 2010;12: 75–78. | [70] |
| Guimarães M, Hurtado R, Bello C, Vanstreels R, Ferreira A. Surveillance for Newcastle disease virus, avian influenza virus and *Mycoplasma gallisepticum* in wild birds near commercial poultry farms surrounded by Atlantic Rainforest Remnants, Southeastern Brazil. Rev Bras Ciência Avícola. 2016;18: 387–394. doi:10.1590/1806-9061-2015-0164 | [75] |
| Hagen CA, Crupper SS, Applegate RD, Robel RJ. Prevalence of mycoplasma antibodies in lesser prairie-chicken sera. Avian Dis. 2002;46: 708–12. doi:10.1637/0005-2086(2002)046[0708:POMAIL]2.0.CO;2 | [61] |
| Hartup BK, Kollias G V. Field Investigation of *Mycoplasma gallisepticum* Infections in House Finch (*Carpodacus mexicanus*) Eggs and Nestlings. Avian Dis. 1999;43: 572. doi:10.2307/1592658 | [85] |
| Hensley TS, Cain JR. Prevalence of certain antibodies to selected disease-causing agents in wild turkeys in Texas. Avian Dis. 1979;23: 62–69. | [8] |
| Hernandez SM, Peters VE, Weygandt PL, Jimenez C, Villegas P, O’Connor B, et al. Do shade-grown coffee plantations pose a disease risk for wild birds? Ecohealth. 2013;10: 145–158. doi:10.1007/s10393-013-0837-3 | [76] |
| Hoffman RW, Page Luttrell M, Davidson WR, Ley DH. Mycoplasmas in wild turkeys living in association with domestic fowl. J Wildl Dis. 1997;33: 526–535. doi:10.7589/0090-3558-33.3.526 | [57] |
| Hopkins BA, Skeeles JK, Houghten GE, Slagle D, Gardner K. A survey of infectious diseases in wild turkeys (*Meleagridis gallopavo silvestris*) from Arkansas. J Wildl Dis. 1990;26: 468–472. | [79] |
| Ley DH, Berkhoff JE, Mclaren JM. *Mycoplasma gallisepticum* isolated from house finches (*Carpodacus mexicanus*) with conjunctivitis. Avian Dis. 1996;40: 480–483. doi:10.2307/1592250 | [13] |
| Ley DH, Hawley DM, Geary SJ, Dhondt AA. House finch (*Haemorhous mexicanus*) conjunctivitis, and *Mycoplasma* spp. isolated from North American wild birds, 1994–2015. J Wildl Dis. 2016;52: 669–673. doi:10.7589/2015-09-244 | [20] |
| Lierz M, Hangen N, Hernandez-Divers J, Hafez HM. Occurrence of mycoplasmas in semen samples of birds of prey. Avian Pathol. 2008;37: 495–497. doi:10.1080/03079450802356961 | [74] |
| Lierz M, Schmidt R, Runge M. *Mycoplasma* species isolated from falcons in the Middle East. Vet Rec. 2002;151: 92–93. doi:10.1136/vr.151.3.92 | [73] |
| Luttrell MP, Eleazer TH, Kleven SH. *Mycoplasma gallopavonis* in Eastern wild turkeys. J Wildl Dis. 1992;28: 288–291. doi:10.7589/0090-3558-28.2.288 | [59] |
| Luttrell MP, Fischer JR, Stallknecht DE, Kleven SH. Field investigation of *Mycoplasma gallisepticum* infections in house finches (*Carpodacus mexicanus*) from Maryland and Georgia. Avian Dis. 1996;40: 335. doi:10.2307/1592229 | [27] |
| Luttrell MP, Kleven SH, Davidson WR. An investigation of the persistence of *Mycoplasma* *gallisepticum* in an Eastern population of wild turkeys. J Wildl Dis. 1991;27: 74–80. doi:10.7589/0090-3558-27.1.74 | [58] |
| Luttrell MP, Kleven SH, Mahnke GM. *Mycoplasma synoviae* in a released pen-raised wild turkey. Avian Dis. 1992;36: 169. doi:10.2307/1591734 | [60] |
| Luttrell MP, Stallknecht DE, Fischer JR, Sewell CT, Kleven SH. Natural *Mycoplasma gallisepticum* infection in a captive flock of house finches. J Wildl Dis. 1998;34: 289–296. doi:10.7589/0090-3558-34.2.289 | [14] |
| Luttrell MP, Stallknecht DE, Kleven SH, Kavanaugh DM, Corn JL, Fischer JR. *Mycoplasma gallisepticum* in house finches (*Carpodacus mexicanus*) and other wild birds associated with poultry production facilities. Avian Dis. 2001;45: 321. doi:10.2307/1592971 | [65] |
| Marques MVR, Junior FCF, de Assis Andery D, Fernandes AA, de Araújo AV, de Resende JS, et al. Health assessment of captive tinamids (*Aves, Tinamiformes*) in Brazil. J Zoo Wildl Med. 2012;43: 539–548. doi:10.1638/2011-0262R1.1 | [72] |
| Michiels T, Welby S, Vanrobaeys M, Quinet C, Rouffaer L, Lens L, et al. Prevalence of *Mycoplasma gallisepticum* and *Mycoplasma synoviae* in commercial poultry, racing pigeons and wild birds in Belgium. Avian Pathol. 2016;45: 244–252. doi:10.1080/03079457.2016.1145354 | [46] |
| Mikaelian I, Ley DH, Claveau R, Lemieux M, Bérubé J-P. *Mycoplasmosis* in evening and pinegrosbeaks with conjunctivitis in Quebec. J Wildl Dis. 2001;37: 826–830. doi:10.7589/0090-3558-37.4.826 | [62] |
| Morishita TY, Aye PP, Ley EC, Harr BS. Survey of pathogens and blood parasites in free-living passerines. Avian Dis. 1999;43: 549. doi:10.2307/1592655 | [82] |
| Morishita TY, McFadzen ME, Mohan R, Aye PP, Brooks DL. Serologic survey of free-living nestling prairie falcons (*Falco mexicanus*) for selected pathogens. J Zoo Wildl Med. 1998;29: 18–20. doi:10.2307/1592655 | [48] |
| Parsons NJ, Gous TA, Schaefer AM, Vanstreels RET. Health evaluation of African penguins (*Spheniscus demersus*) in southern Africa. Onderstepoort J Vet Res. 2016;83: 1–13. doi:10.4102/ojvr.v83i1.1147 | [71] |
| Pennycott TW, Dare CM, Yavari CA, Bradbury JM. *Mycoplasma sturni* and *Mycoplasma gallisepticum* in wild birds in Scotland. Vet Rec. 2005;156: 513–515. doi:10.1136/vr.156.16.513 | [64] |
| Peterson MJ, Aguirre R, Ferro PJ, Jones DA, Lawyer TA, Peterson MN, et al. Infectious disease survey of Rio Grande wild turkeys in the Edwards plateau of Texas. J Wildl Dis. 2002;38: 826–833. doi:10.7589/0090-3558-38.4.826 | [50] |
| Poveda JB, Carranza J, Miranda A, Garrido A, Hermoso M, Fernandez A, et al. An epizootiological study of avian mycoplasmas in Southern Spain. Avian Pathol. 1990;19: 627–633. doi:10.1080/03079459008418718 | [47] |
| Roberts SR, Nolan PM, Lauerman LH, Li L-Q, Hill GE. Characterization of the mycoplasmal conjunctivitis epizootic in a house finch population in the southeastern USA. J Wildl Dis. 2001;37: 82–88. doi:10.7589/0090-3558-37.1.82 | [68] |
| Sasseville VG, Miller B, Nielsen SW. A pathologic study of wild turkeys in Connecticut. Cornell Vet. 1988;78: 353–64. | [80] |
| Shimizu T, Numano K, Uchida K. Isolation and identification of mycoplasmas from various birds: an ecological study. Japanese J Vet Sci. 1979;41: 273–282. doi:10.1292/jvms1939.41.273 | [10] |
| Soos C, Padilla L, Iglesias A, Gottdenker N, Bedon MC, Rios A, et al. Comparison of pathogens in broiler and backyard chickens on the Galápagos Islands: Implications for transmission to wildlife. Auk. 2008;125: 445–455. doi:10.1525/auk.2008.06235 | [83] |
| Staley M, Bonneaud C, McGraw KJ, Vleck CM, Hill GE. Detection of *Mycoplasma gallisepticum* in House Finches (*Haemorhous mexicanus*) from Arizona. Avian Dis. 2018;62: 14–17. doi:10.1637/11610-021317-reg.1 | [66] |
| Stallknecht DE, Johnson DC, Emory WH, Kleven SH. Wildlife surveillance during a *Mycoplasma gallisepticum* epornitic in domestic turkeys. Avian Dis. 1982;26: 883–890. | [69] |
| Straub MH, Kelly TR, Rideout BA, Eng C, Wynne J, Braun J, et al. Seroepidemiologic survey of potential pathogens in obligate and facultative scavenging avian species in California. Margalida A, editor. PLoS One. 2015;10: e0143018. doi:10.1371/journal.pone.0143018 | [45] |
| Trainer DO, Glazener WC, Hanson RP, Nassif BD. Infectious disease exposure in a wild turkey population. Avian Dis. 1968;12: 208–214. doi:10.2307/1588102 | [9] |
| Veatch JK, Applegate RD, Osborne SJ. Serologic incidence of some diseases in Kansas wild turkeys. Avian Dis. 1998;42: 393. doi:10.2307/1592492 | [51] |
| Wellehan JFX, Calsamiglia M, Ley DH, Zens MS, Amonsin A, Kapur V. Mycoplasmosis in captive crows and robins from Minnesota. J Wildl Dis. 2001;37: 547–555. doi:10.7589/0090-3558-37.3.547 | [52] |
| Williams CK, Davidson WR, Lutz RS, Applegate RD. Health Status of Northern Bobwhite Quail (*Colinus virginianus*) in Eastern Kansas. Avian Dis. 2000;44: 953. doi:10.2307/1593071 | [81] |
