# Supplementary material for: Occurrence of Mycoplasma gallisepticum in wild birds: A systematic review and meta-analysis
Source: PLoS One. 2020 Apr 16;15(4):e0231545. doi: 10.1371/journal.pone.0231545 (PMC7162529; doi:10.1371/journal.pone.0231545)
Supplement: S3 Table — (DOCX) [file pone.0231545.s004.docx]

S4 Table. Number of studies by region and country.

| **Region** | **Country** | **No of studies** |
| --- | --- | --- |
| Africa | Namibia and South Africa | 1 |
| Asia | Japan | 1 |
|  | Malaysia | 1 |
|  | United Arab Emirates | 1 |
| Europe | Belgium | 1 |
|  | Germany | 1 |
|  | Spain | 1 |
|  | UK | 1 |
| North America | Canada | 1 |
|  | Costa Rica | 1 |
|  | Mexico | 1 |
|  | USA | 35 |
| South America | Brazil | 4 |
|  | Galápagos Islands | 2 |
