# Supplementary material for: Occurrence of Mycoplasma gallisepticum in wild birds: A systematic review and meta-analysis
Source: PLoS One. 2020 Apr 16;15(4):e0231545. doi: 10.1371/journal.pone.0231545 (PMC7162529; doi:10.1371/journal.pone.0231545)
Supplement: S4 Table — (DOCX) [file pone.0231545.s005.docx]

S5 Table. Number of studies by order and species of wild birds.

| **Order** | **Family** | **Species** | **No of studie**s |
| --- | --- | --- | --- |
| *Accipitriformes* | *Accipitridae* | Northern goshawk (*Accipiter gentilis*) | 1 |
|  |  | golden eagle (*Aquila chrysaetos*) | 1 |
|  |  | white-tailed hawk (*Buteo albicaudatus*) | 1 |
|  |  | short-tailed hawk (*Buteo brachyurus*) | 1 |
|  |  | common buzzard (*Buteo buteo*) | 2 |
|  |  | Western marsh harrier (*Circus aeruginosus*) | 1 |
|  |  | griffon vulture (*Gyps fulvus*) | 1 |
|  |  | savanna hawk (*Heterospizias meridionalis*) | 1 |
|  |  | gray-headed kite (*Leptodon cayanensis*) | 1 |
|  |  | red kite (*Milvus migrans*) | 1 |
|  |  | roadside hawk (*Rupornis magnirostris*) | 1 |
|  |  | mountain hawk-eagle (*Spizaetus nipalensis*) | 1 |
|  | *Cathartidae* | turkey vulture (*Cathartes aura*) | 2 |
|  |  | black vulture (*Coragyps atratus*) | 1 |
|  |  | California condor (*Gymnogyps californianus*) | 1 |
| *Anseriformes* | *Anatidae* | northern shoveler (*Anas clypeata*) | 2 |
|  |  | common teal (*Anas crecca*) | 2 |
|  |  | Mexican duck (*Anas diazi*) | 1 |
|  |  | falcated teal (*Anas falcata*) | 1 |
|  |  | mallard (*Anas platyrhynchos*) | 3 |
|  |  | greylag goose (*Anser anser*) | 1 |
|  |  | common pochard (*Aythya ferina*) | 1 |
|  |  | Bewick's swan (*Cygnus bewickii*) | 1 |
|  |  | mute swan (*Cygnus olor*) | 1 |
|  |  | black-bellied whistling duck (*Dendrocygna autumnalis*) | 1 |
|  |  | fulvous whistling duck (*Dendrocygna bicolor*) | 1 |
| *Apodiformes* | *Trochilidae* | glittering-bellied emerald (*Chlorostilbon aureoventris*) | 1 |
|  |  | saw-billed hermit (*Ramphodon naevius*) | 1 |
| *Bucerotiformes* | *Upupidae* | hoopoe (*Upupa epops*) | 1 |
| *Charadriiformes* | *Charadriidae* | killdeer (*Charadrius vociferus*) | 1 |
|  | *Laridae* | herring gull (*Larus argentatus*) | 1 |
|  | *Scolopacidae* | Eurasian woodcock (*Scolopax rusticola*) | 1 |
| *Ciconiformes* | *Ciconidae* | greater adjutant (*Leptoptilos dubius*) | 1 |
| *Columbiformes* | *Columbidae* | domestic pigeon (*Columba livia*) | 1 |
|  |  | common wood pigeon (*Columba palumbus*) | 2 |
|  |  | Mexican dove (*Columbina inca*) | 1 |
|  |  | ruddy ground dove (*Columbina talpacoti*) | 1 |
|  |  | white-tipped dove (*Leptotila verreauxi*) | 1 |
|  |  | Eurasian collared dove (*Streptopelia decaocto*) | 1 |
|  |  | white-winged dove (*Zenaida asiatica*) | 1 |
|  |  | mourning dove (*Zenaida macroura*) | 2 |
| *Cuculiformes* | *Cuculidae* | groove-billed ani (*Crotophaga sulcirostris*) | 1 |
| *Coraciiformes* | *Momotidae* | blue-crowned motmot (*Momotus momota*) | 1 |
| *Cuculiformes* | *Cuculidae* | smooth-billed ani (*Crotophaga ani*) | 1 |
| *Falconiformes* | *Falconidae* | southern crested caracara (*Caracara plancus*) | 1 |
|  |  | lanner falcon (*Falco biarmicus*) | 1 |
|  |  | saker falcon (*Falco cherrug*) | 1 |
|  |  | aplomado falcon (*Falco femoralis*) | 1 |
|  |  | prairie falcon (*Falco mexicanus*) | 1 |
|  |  | barbary falcon (*Falco peregrinoides*) | 1 |
|  |  | peregrine falcon (*Falco peregrinus*) | 2 |
|  |  | bat falcon (*Falco rufigularis*) | 1 |
|  |  | gyr falcon (*Falco rusticolus*) | 1 |
|  |  | American kestrel (*Falco sparverius*) | 1 |
|  |  | Eurasian hobby (*Falco subbuteo*) | 1 |
|  |  | common kestrel (*Falco tinnunculus*) | 2 |
|  |  | yellow-headed caracara (*Milvago chimachima*) | 1 |
| *Galliformes* | *Odontophoridae* | scaled quail (*Callipepla squamata*) | 1 |
|  |  | Northern bobwhite (*Colinus virginianus*) | 3 |
|  | *Phasianidae* | Chinese bamboo partridge *(Bambusicola thoracica*) | 1 |
|  |  | common quail (*Coturnix coturnix*) | 1 |
|  |  | wild turkey (*Meleagris gallopavo*) | 18 |
|  |  | Indian peafowl (*Pavo cristatus*) | 1 |
|  |  | green peafowl (*Pavo muticus*) | 1 |
|  |  | grey partridge (*Perdix perdix*) | 1 |
|  |  | common pheasant (*Phasianus colchicus*) | 2 |
|  |  | copper pheasant (*Phasianus soemmerringii*) | 1 |
|  |  | lesser prairie-chicken (*Tympanuchius pallidicinctus*) | 1 |
| *Gruiformes* | *Gruidae* | demoisella crane (*Anthropoides virgo*) | 1 |
|  |  | white crowned crane (*Balearica pavonina*) | 1 |
|  | *Rallidae* | American coot (*Fulica americana*) | 1 |
|  |  | common coot (*Fulica atra*) | 3 |
|  |  | watercock (*Gallicrex cinerea*) | 1 |
|  |  | common moorhen (*Gallinula chloropus*) | 1 |
| *Passeriformes* | *Bombycillidae* | cedar waxwing (*Bombycilla garrulus*) | 1 |
|  | *Cardinalidae* | northern cardinal (*Cardinalis cardinalis*) | 3 |
|  |  | red-crowned ant tanager (*Habia rubica*) | 1 |
|  | *Corvidae* | western scrub-jay (*Aphelocoma californica*) | 2 |
|  |  | American crow (*Corvus brachyrhynchos*) | 3 |
|  |  | carrion crow (*Corvus corone*) | 2 |
|  |  | rook (*Corvus frugilegus*) | 1 |
|  |  | large-billed crow (*Corvus macrorhynchos*) | 1 |
|  |  | jackdaw (*Corvus monedula*) | 2 |
|  |  | house crow (*Corvus splendens*) | 1 |
|  |  | blue jay (*Cyanocitta cristata*) | 3 |
|  |  | Eurasian jay (*Garrulus glandarius*) | 1 |
|  |  | Eurasian magpie (*Pica pica*) | 2 |
|  |  | brown jay (*Psilorhinus morio*) | 2 |
|  | *Estrildidae* | white-rumped munia (*Lonchura striata*) | 1 |
|  | *Fringillidae* | pine siskin (*Carduelis pinus*) | 1 |
|  |  | greenfinch (*Chloris chloris*) | 1 |
|  |  | evening grosbeak (*Coccothraustes vespertinus*) | 3 |
|  |  | yellow-throated euphonia (*Euphonia hirundinacea*) | 1 |
|  |  | house finch (*Haemorhous mexicanus*) | 12 |
|  |  | purple finch (*Haemorhous purpureus*) | 5 |
|  |  | pine grosbeak (*Pinicola enucleator*) | 1 |
|  |  | lesser goldfinch (*Spinus psaltria*) | 2 |
|  |  | Eurasian siskin (*Spinus spinus*) | 1 |
|  |  | American goldfinch (*Spinus tristis*) | 7 |
|  | *Furnariidae* | ruddy woodcreeper (*Dendrocincla homochroa*) | 1 |
|  |  | northern barred woodcreeper (*Dendrocolaptes sanctithomae*) | 1 |
|  |  | rufous hornero (*Furnarius rufus*) | 1 |
|  |  | streak-headed woodcreeper (*Lepidocolaptes souleyetii*) | 1 |
|  | *Icteridae* | red-winged blackbird (*Agelaius phoeniceus*) | 2 |
|  |  | chestnut-capped blackbird (*Chrysomus ruficapillus*) | 1 |
|  |  | yellow-breasted chat (*Icteria virens*) | 2 |
|  |  | Baltimore oriole (*Icterus galbula*) | 1 |
|  |  | bronzed cowbird (*Molothrus aeneus*) | 1 |
|  |  | brown-headed cowbird (*Molothrus ater*) | 6 |
|  |  | shiny cowbird (*Molothrus bonariensis*) | 1 |
|  |  | Mexican grackle (*Quiscalus mexicanus*) | 1 |
|  |  | common grackle (*Quiscalus quiscula*) | 2 |
|  |  | eastern meadowlark (*Sturnella magna*) | 1 |
|  | *Mimidae* | gray catbird (*Dumetella carolinensis*) | 2 |
|  |  | northern mockingbird (*Mimus polyglottos*) | 2 |
|  |  | Floreana mockingbird (*Mimus trifasciatus*) | 1 |
|  |  | Galápagos mockingbird (*Nesomimus parvulus*) | 1 |
|  |  | brown thrasher (*Toxostoma rufum*) | 2 |
|  | *Muscicapidae* | Siberian blue robin (*Larvivora cyane*) | 1 |
|  | *Paridae* | tufted titmouse (*Baeolophus bicolor*) | 4 |
|  |  | black-capped chickadee (*Poecile atricapillus*) | 3 |
|  |  | Carolina chickadee (*Poecile carolinensis*) | 2 |
|  | *Parulidae* | striped-crowned warbler (*Basileuterus culicivorus*) | 1 |
|  |  | rufous-capped warbler (*Basileuterus rufifrons*) | 1 |
|  |  | myrtle warbler (*Dendroica coronata*) | 1 |
|  |  | yellow warbler (*Dendroica petechia*) | 1 |
|  |  | common yellowthroat (*Geothlypis trichas*) | 1 |
|  |  | yellow-rumped warbler (*Setophaga coronata*) | 1 |
|  |  | pine warbler (*Setophaga pinus*) | 1 |
|  | *Passerellidae* | white-naped brush finch (*Atlapetes albinucha*) | 1 |
|  |  | dark-eyed junco (*Junco hyemalis*) | 2 |
|  |  | swamp sparrow (*Melospiza georgiana*) | 1 |
|  |  | song sparrow (*Melospiza melodia*) | 4 |
|  |  | white-eared ground sparrow (*Melozone leucotis*) | 1 |
|  |  | Eastern towhee (*Pipilo erythrophthalmus*) | 1 |
|  |  | American tree sparrow (*Spizella arborea*) | 2 |
|  |  | chipping sparrow (*Spizella passerina*) | 2 |
|  |  | white-throated sparrow (*Zonotrichia albicollis*) | 4 |
|  |  | rufous-collared sparrow (*Zonotrichia capensis*) | 1 |
|  |  | white-crowned sparrow (*Zonotrichia leucophrys*) | 1 |
|  | *Passeridae* | house sparrow (*Passer domesticus*) | 8 |
|  |  | Eurasian tree sparrow (*Passer montanus*) | 1 |
|  | *Pipridae* | long-tailed manakin (*Chiroxiphia linearis*) | 1 |
|  | *Pycnonotidae* | brown-eared bulbul (*Hypsipetes amaurotis*) | 1 |
|  | *Regulidae* | ruby-crowned kinglet (*Regulus calendula*) | 1 |
|  |  | golden-crowned kinglet (*Regulus satrapa*) | 1 |
|  | *Sittidae* | white-breasted nuthatch (*Sitta carolinensis*) | 1 |
|  | *Sturnidae* | common hill myna (*Gracula religiosa*) | 1 |
|  |  | common starling (*Sturnus vulgaris*) | 5 |
|  | *Thraupidae* | vegetarian finch (*Camarhynchus crassirostris*) | 1 |
|  |  | woodpecker finch (*Camarhynchus pallidus*) | 1 |
|  |  | small tree finch (*Camarhynchus parvulus*) | 1 |
|  |  | green warbler-finch (*Certhidea olivacea*) | 1 |
|  |  | medium ground finch (*Geospiza fortis*) | 1 |
|  |  | small ground finch (*Geospiza fuliginosa*) | 1 |
|  |  | greyish saltator (*Saltator coerulescens*) | 1 |
|  |  | buff-throated saltator (*Saltator maximus*) | 1 |
|  |  | saffron finch (*Sicalis flaveola*) | 1 |
|  |  | ruby-crowned tanager (*Tachyphonus coronatus*) | 1 |
|  |  | blue-gray tanager (*Thraupis episcopus*) | 1 |
|  |  | sayaca tanager (*Thraupis sayaca*) | 1 |
|  | *Thyrannidae* | yellow-bellied elaenia (*Elaenia flavogaster*) | 1 |
|  |  | masked water-tyrant (*Fluvicola nengeta*) | 1 |
|  |  | boat-billed flycatcher (*Megarynchus pitangua*) | 1 |
|  |  | great kiskadee (*Pitangus sulphuratus*) | 1 |
|  |  | Galápagos flycatcher (*Myiarchus magnirostris*) | 2 |
|  |  | common tody-flycatcher (*Todirostrum cinereum*) | 1 |
|  | *Troglodytidae* | rufous-and-white wren (*Thryophilus rufalbus*) | 1 |
|  |  | Carolina wren (*Thryothorus ludovicianus*) | 1 |
|  |  | house wren (*Troglodytes aedon*) | 2 |
|  | *Turdidae* | orange-billed nightingale-thrush (*Catharus aurantiirostris*) | 1 |
|  |  | hermit thrush (*Catharus guttatus*) | 1 |
|  |  | Swainson's thrush (*Catharus ustulatus*) | 1 |
|  |  | wood thrush (*Hylocichla mustelina*) | 1 |
|  |  | creamy-bellied thrush (*Turdus amaurochalinus*) | 1 |
|  |  | white-throated thrush (*Turdus assimilis*) | 1 |
|  |  | yellow-legged thrush (*Turdus flavipes*) | 1 |
|  |  | clay-colored thrush (*Turdus grayi*) | 1 |
|  |  | common blackbird (*Turdus merula*) | 1 |
|  |  | American robin (*Turdus migratorius*) | 5 |
|  |  | dusky thrush (*Turdus naumanii*) | 1 |
|  |  | rufous-bellied thrush (*Turdus rufivenaris*) | 1 |
| *Pelecaniformes* | *Ardeidae* | grey heron (*Ardea cinerea*) | 1 |
|  |  | purple heron (*Ardea purpurea*) | 1 |
|  |  | cattle egret (*Bubulcus ibis*) | 1 |
|  |  | little egret (*Egretta garzetta*) | 1 |
|  |  | tricolored heron (*Egretta tricolor*) | 1 |
|  |  | black-crowned night heron (*Nycticorax nycticorax*) | 1 |
| *Phoenicopteriformes* | *Phoenicopteridae* | greater flamingo (*Phoenicopterus roseus*) | 1 |
| *Piciformes* | *Picidae* | downy woodpecker (*Picoides pubescens*) | 1 |
|  |  | Japanese green woodpecker (*Picus awokera*) | 1 |
|  | *Ramphastidae* | blue-throated toucanet (*Aulacorhynchus caeruleogularis*) | 1 |
| *Psittaciformes* | *Psittacidae* | Amazon parrot (*Amazona aestiva*) | 1 |
|  |  | orange-winged parrot (*Amazona amazonica*) | 1 |
|  |  | hyacinth macaw (*Anodorhynchus hyacinthinus*) | 1 |
|  |  | blue-and-yellow macaw (*Ara* *ararauna*) | 1 |
|  |  | scarlet macaw (*Ara macao*) | 1 |
|  |  | jandaya parakeet or jandaya conure (*Aratinga jandaya*) | 1 |
|  |  | golden conure or golden parakeet (*Guarouba guarouba*) | 1 |
|  |  | dusky parrot (*Pionus fuscus*) | 1 |
|  | *Psittaculidae* | budgerigar (*Melopsittacus* sp.) | 1 |
|  |  | parkeet (*Psittacula* sp.) | 1 |
| *Sphenisciformes* | *Spheniscidae* | African penguin (*Spheniscus demersus*) | 1 |
| *Strigiformes* | *Strigidae* | striped owl (*Asio clamator*) | 1 |
|  |  | long-eared owl (*Asio otus*) | 1 |
|  |  | stygian owl (*Asio stygius*) | 1 |
|  |  | burrowing owl (*Athene cunicularia*) | 1 |
|  |  | little owl (*Athene noctua*) | 1 |
|  |  | great horned owl (*Bubo virginianus*) | 1 |
|  |  | ferruginous pygmy owl (*Glaucidium brasilianum*) | 1 |
|  |  | tropical screech owl (*Megascops choliba*) | 1 |
|  |  | tawney owl (*Strix aluco*) | 1 |
|  |  | black-banded owl (*Strix huhula*) | 1 |
|  |  | Ural owl (*Strix uralensis*) | 1 |
|  |  | mottled owl (*Strix virgata*) | 1 |
|  | *Tytonidae* | barn owl (*Tyto alba*) | 4 |
| *Struthioniformes* | *Struthionidae* | common ostrich (*Struthio camelus*) | 1 |
| *Tinamiformes* | *Tinamidae* | bown tinamou (*Crypturellus obsoletus*) | 1 |
|  |  | small-billed tinamou (*Crypturellus parvirostris*) | 1 |
|  |  | tataupa tinamou (*Crypturellus tataupa*) | 1 |
|  |  | undulated tinamou (*Crypturellus undulatus*) | 1 |
|  |  | red-winged tinamou (*Rhynchotus rufescens*) | 1 |
|  |  | solitary tinamou (Tinam*us solitarius*) | 1 |
