# Supplementary material for: Occurrence of Mycoplasma gallisepticum in wild birds: A systematic review and meta-analysis
Source: PLoS One. 2020 Apr 16;15(4):e0231545. doi: 10.1371/journal.pone.0231545 (PMC7162529; doi:10.1371/journal.pone.0231545)
Supplement: S5 Table — (DOCX) [file pone.0231545.s006.docx]

S6 Table. Subgroup meta-analysis of the MG prevalence by culture method.

| **Subgroup** | **No of prevalence inputs** | **Sample size** | **Mean (%)** | **95% CI** | **I^2^ (%)** | **Difference between groups** |
| --- | --- | --- | --- | --- | --- | --- |
| **Country** | | | | | | p<0.0001 |
| Canada | 1 | 5 | 20 | 0.1 - 61.6 |  |  |
| Germany | 1 | 40 | 0 | 0 - 2.4 |  |  |
| Japan | 1 | 217 | 2.8 | 1 - 5.4 |  |  |
| Malaysia | 1 | 148 | 29.7 | 22.7 - 37.3 |  |  |
| Spain | 1 | 80 | 2.5 | 0.2 - 7 |  |  |
| UK | 1 | 41 | 0 | 0 - 2.3 |  |  |
| USA | 16 | 2639 | 17.3 | 1.5 - 45.3 | 99.6 |  |
| **Region** | | | | | | p=0.0571 |
| Asia | 3 | 385 | 6.5 | 0 - 30.1 | 97 |  |
| Europe | 3 | 161 | 0.4 | 0 - 3.1 | 50.7 |  |
| North America | 17 | 2644 | 17.5 | 1.7 - 44.6 | 99.5 |  |
| **Species** | | | | | | p<0.0001 |
|  |  |  |  |  |  |  |
| *Accipiter gentilis* | 1 | 13 | 0 | 0 - 7.2 |  |  |
| *Agelaius phoeniceus* | 1 | 1 | 0 | 0 - 69 |  |  |
| *Anas clypeata* | 2 | 4 | 0 | 0 - 22.2 | 0 |  |
| *Anas crecca* | 2 | 11 | 0 | 0 - 8.5 | 0 |  |
| *Anas falcata* | 1 | 2 | 0 | 0 - 40.8 |  |  |
| *Anas platyrhynchos* | 2 | 27 | 0 | 0 - 3.5 | 0 |  |
| *Anthropoides virgo* | 1 | 1 | 0 | 0 - 69 |  |  |
| *Aphelocoma californica* | 1 | 1 | 100 | 31 - 100 |  |  |
| *Ara macao* | 1 | 4 | 0 | 0 - 22.2 |  |  |
| *Ardea purpurea* | 1 | 2 | 0 | 0 - 40.8 |  |  |
| *Aythya ferina* | 1 | 4 | 0 | 0 - 22.2 |  |  |
| *Baeolophus bicolor* | 2 | 45 | 0 | 0 - 2.1 | 0 |  |
| *Balearica pavonina* | 1 | 1 | 0 | 0 - 69 |  |  |
| *Bambusicola thoracica* | 1 | 8 | 0.0 | 0 - 11.5 |  |  |
| *Buteo buteo* | 1 | 7 | 0 | 0 - 13.1 |  |  |
| *Cardinalis cardinalis* | 2 | 55 | 0 | 0 - 1.7 | 0 |  |
| *Catharus guttatus* | 1 | 3 | 0 | 0 - 28.7 |  |  |
| *Chloris chloris* | 1 | 2 | 0 | 0 - 40.8 |  |  |
| *Circus aeruginosus* | 1 | 8 | 0 | 0 - 11.5 |  |  |
| *Coccothraustes vespertinus* | 2 | 4 | 85 | 15.8 - 100 | 59.5 |  |
| *Colinus virginianus* | 1 | 7 | 0 | 0 - 13.1 |  |  |
| *Columba palumbus* | 1 | 2 | 0 | 0 - 40.8 |  |  |
| *Corvus brachyrhynchos* | 1 | 2 | 50 | 0.9 - 99.2 |  |  |
| *Corvus corone* | 1 | 6 | 0 | 0 - 15.2 |  |  |
| *Corvus frugilegus* | 1 | 13 | 0 | 0 - 7.2 |  |  |
| *Corvus macrorhynchos* | 1 | 1 | 0 | 0 - 69 |  |  |
| *Corvus monedula* | 1 | 1 | 0 | 0 - 69 |  |  |
| *Corvus splendens* | 1 | 148 | 30 | 22.7 - 37.3 |  |  |
| *Cyanocitta cristata* | 3 | 6 | 43 | 0 - 100 | 75.7 |  |
| *Cygnus bewickii* | 1 | 5 | 0 | 0 - 18 |  |  |
| *Dendroica coronata* | 1 | 27 | 0 | 0 - 3.5 |  |  |
| *Dumetella carolinensis* | 1 | 2 | 0 | 0 - 40.8 |  |  |
| *Egretta garzetta* | 1 | 1 | 0 | 0 - 69 |  |  |
| *Falco biarmicus* | 1 | 2 | 0 | 0 - 40.8 |  |  |
| *Falco cherrug* | 1 | 9 | 0.0 | 0 - 10.3 |  |  |
| *Falco peregrinoides* | 1 | 2 | 0 | 0 - 40.8 |  |  |
| *Falco peregrinus* | 2 | 7 | 48 | 0 - 100 | 92.9 |  |
| *Falco rusticolus* | 1 | 2 | 0 | 0 - 40.8 |  |  |
| *Falco subbuteo* | 1 | 1 | 0.0 | 0 - 69 |  |  |
| *Falco tinnunculus* | 1 | 5 | 0 | 0 - 18 |  |  |
| *Fulica atra* | 2 | 11 | 0 | 0 - 8.5 | 0 |  |
| *Gallicrex cinerea* | 1 | 1 | 0 | 0 - 69 |  |  |
| *Gallinula chloropus* | 1 | 3 | 0 | 0 - 28.7 |  |  |
| *Gracula religiosa* | 1 | 3 | 0 | 0 - 28.7 |  |  |
| *Gyps fulvus* | 1 | 2 | 0 | 0 - 40.8 |  |  |
| *Haemorhous mexicanus* | 6 | 524 | 41 | 3.7 - 86.9 | 99.2 |  |
| *Haemorhous purpureus* | 1 | 3 | 100 | 71.3 - 100 |  |  |
| *Hypsipetes amaurotis* | 1 | 1 | 0 | 0 - 69 |  |  |
| *Icteria virens* | 1 | 2 | 0 | 0 - 40.8 |  |  |
| *Larvivora cyane* | 1 | 5 | 0 | 0 - 18 |  |  |
| *Leptoptilos dubius* | 1 | 2 | 0 | 0 - 40.8 |  |  |
| *Lonchura striata var. domestica* | 1 | 3 | 0 | 0 - 28.7 |  |  |
| *Meleagris gallopavo* | 8 | 912 | 12 | 0 - 59 | 99.4 |  |
| *Melopsittacus sp.* | 1 | 2 | 0 | 0 - 40.8 |  |  |
| *Melospiza georgiana* | 1 | 1 | 0 | 0 - 69 |  |  |
| *Melospiza melodia* | 1 | 3 | 0 | 0 - 28.7 |  |  |
| *Milvus migrans* | 1 | 5 | 0 | 0 - 18 |  |  |
| *Mimus polyglottos* | 2 | 12 | 0 | 0 - 7.8 | 0 |  |
| *Molothrus ater* | 3 | 184 | 0 | 0 - 0.5 | 0 |  |
| *Nycticorax nycticorax* | 1 | 9 | 0 | 0 - 10.3 |  |  |
| *Passer domesticus* | 4 | 393 | 0 | 0 - 0.2 | 0 |  |
| *Passer montanus* | 1 | 94 | 6 | 2.4 - 12.2 |  |  |
| *Pavo cristatus* | 1 | 1 | 0 | 0 - 69 |  |  |
| *Phasianus colchicus* | 1 | 21 | 0 | 0 - 4.5 |  |  |
| *Phasianus soemmerringii* | 1 | 4 | 0 | 0 - 22.2 |  |  |
| *Phoenicopterus roseus* | 1 | 2 | 0 | 0 - 40.8 |  |  |
| *Pica pica* | 1 | 3 | 0 | 0 - 28.7 |  |  |
| *Picus awokera* | 1 | 1 | 0 | 0 - 69 |  |  |
| *Pinicola enucleator* | 1 | 3 | 0 | 0 - 28.7 |  |  |
| *Pipilo erythrophthalmus* | 1 | 7 | 0 | 0 - 13.1 |  |  |
| *Poecile atricapillus* | 1 | 1 | 0 | 0 - 69 |  |  |
| *Poecile carolinensis* | 2 | 18 | 0 | 0 - 5.2 | 0 |  |
| *Psittacula sp.* | 1 | 6 | 0 | 0 - 15.2 |  |  |
| *Quiscalus quiscula* | 2 | 143 | 0 | 0 - 0.7 | 0 |  |
| *Regulus calendula* | 1 | 9 | 0.0 | 0 - 10.3 |  |  |
| *Regulus satrapa* | 1 | 5 | 0 | 0 - 18 |  |  |
| *Scolopax rusticola* | 1 | 7 | 0 | 0 - 13.1 |  |  |
| *Setophaga coronata* | 1 | 1 | 0 | 0 - 69 |  |  |
| *Setophaga pinus* | 1 | 4 | 0 | 0 - 22.2 |  |  |
| *Spinus psaltria* | 1 | 2 | 100 | 59.2 - 100 |  |  |
| *Spinus spinus* | 1 | 1 | 0 | 0 - 69 |  |  |
| *Spinus tristis* | 3 | 51 | 24 | 0 - 98 | 95.5 |  |
| *Spizaetus nipalensis* | 1 | 2 | 0 | 0 - 40.8 |  |  |
| *Spizella passerina* | 2 | 21 | 0.0 | 0 - 4.5 | 0 |  |
| *Streptopelia decaocto* | 1 | 1 | 0 | 0 - 69 |  |  |
| *Strix uralensis* | 1 | 1 | 0 | 0 - 69 |  |  |
| *Struthio camelus* | 1 | 1 | 0 | 0 - 69 |  |  |
| *Sturnella magna* | 1 | 24 | 0 | 0 - 4 |  |  |
| *Sturnus vulgaris* | 3 | 103 | 0 | 0 - 0.9 | 0 |  |
| *Thryothorus ludovicianus* | 1 | 6 | 0 | 0 - 15.2 |  |  |
| *Toxostoma rufum* | 2 | 10 | 0 | 0 - 9.3 | 0 |  |
| *Troglodytes aedon* | 1 | 1 | 0 | 0 - 69 |  |  |
| *Turdus merula* | 1 | 3 | 0 | 0 - 28.7 |  |  |
| *Turdus migratorius* | 2 | 5 | 0.0 | 0 - 18 | 0 |  |
| *Turdus naumanii* | 1 | 1 | 0 | 0 - 69 |  |  |
| *Tyto alba* | 1 | 1 | 0 | 0 - 69 |  |  |
| *Upupa epops* | 1 | 1 | 0 | 0 - 69 |  |  |
| *Zenaida macroura* | 2 | 62 | 0.0 | 0 - 1.5 | 0 |  |
| *Zonotrichia albicollis* | 2 | 30 | 0 | 0 - 3.2 | 0 |  |
| **Order** | | | | | | p=0.8537 |
| *Accipitriformes* | 3 | 37 | 0 | 0 - 2.6 | 0.0 |  |
| *Anseriformes* | 2 | 53 | 0 | 0 - 1.8 | 0.0 |  |
| *Bucerotiformes* | 1 | 1 | 0 | 0 - 69 |  |  |
| *Charadriiformes* | 1 | 7 | 0 | 0 - 13.1 |  |  |
| *Ciconiformes* | 1 | 2 | 0 | 0 - 40.8 |  |  |
| *Columbiformes* | 3 | 65 | 0 | 0 - 1.5 | 0.0 |  |
| *Falconiformes* | 3 | 28 | 18 | 0 - 83 | 89.1 |  |
| *Galliformes* | 10 | 953 | 7.6 | 0 - 45.3 | 99.3 |  |
| *Gruiformes* | 2 | 17 | 0 | 0 - 5.5 | 0.0 |  |
| *Passeriformes* | 13 | 1997 | 15.8 | 1.2 - 42.1 | 99.4 |  |
| *Pelecaniformes* | 1 | 12 | 0 | 0 - 7.8 |  |  |
| *Phoenicopteriformes* | 1 | 2 | 0 | 0 - 40.8 |  |  |
| *Piciformes* | 1 | 1 | 0 | 0 - 69 |  |  |
| *Psittaciformes* | 1 | 12 | 0 | 0 - 7.8 |  |  |
| *Strigiformes* | 2 | 2 | 0 | 0 - 40.8 | 0.0 |  |
| *Struthioniformes* | 1 | 1 | 0 | 0 - 69 |  |  |
| **Wild versus captive** | | | | | | p=0.3566 |
| captive | 3 | 63 | 2.43 | 0 - 19.1 | 76.8 |  |
| unknown | 6 | 431 | 18.7 | 3.3 - 42.7 | 96.2 |  |
| wild | 16 | 2696 | 10.7 | 0.1 - 35.7 | 99.6 |  |
