# Supplementary material for: Occurrence of Mycoplasma gallisepticum in wild birds: A systematic review and meta-analysis
Source: PLoS One. 2020 Apr 16;15(4):e0231545. doi: 10.1371/journal.pone.0231545 (PMC7162529; doi:10.1371/journal.pone.0231545)
Supplement: S6 Table — (DOCX) [file pone.0231545.s007.docx]

S7 Table. Subgroup meta-analysis of the MG prevalence by PCR.

| **Subgroup** | **No of prevalence inputs** | **Sample size** | **Mean (%)** | **95% CI** | **I^2^ (%)** | **Difference between groups** |
| --- | --- | --- | --- | --- | --- | --- |
| **Country** | | | | | | p<0.0001 |
| Belgium | 1 | 698 | 0.7 | 0.2 - 1.5 |  |  |
| Brazil | 2 | 241 | 15.3 | 0 - 86.3 | 99.30 |  |
| Canada | 1 | 4 | 100 | 77.9 - 100 |  |  |
| Costa Rica | 1 | 207 | 0 | 0 - 0.5 |  |  |
| Malaysia | 1 | 94 | 4.3 | 1.1 - 9.2 |  |  |
| UK | 1 | 41 | 9.8 | 2.7 - 20.6 |  |  |
| USA | 13 | 3402 | 39.1 | 15.9 - 65.4 | 99.40 |  |
| **Region** | | | | | | p=0.0049 |
| Asia | 1 | 94 | 4.3 | 1.1 - 9.2 |  |  |
| Europe | 2 | 739 | 3.5 | 0 - 16.3 | 88.1 |  |
| North America | 15 | 3613 | 39.2 | 17.7 - 63.1 | 99.4 |  |
| South America | 2 | 241 | 15.3 | 0 - 86.3 | 99.3 |  |
| **Species** | | | | | | p<0.0001 |
| *Agelaius phoeniceus* | 2 | 75 | 4 | 0.8 - 9.5 | 0 |  |
| *Amazona aestiva* | 1 | 59 | 52.5 | 39.9 - 65.1 |  |  |
| *Amazona amazonica* | 1 | 2 | 50 | 0.9 - 99.2 |  |  |
| *Anas platyrhynchos* | 1 | 57 | 1.8 | 0 - 6.7 |  |  |
| *Anodorhynchus hyacinthinus* | 1 | 2 | 0 | 0 - 40.8 |  |  |
| *Anser anser* | 1 | 4 | 0 | 0 - 22.2 |  |  |
| *Aphelocoma californica* | 2 | 2 | 100 | 59.2 - 100 | 0 |  |
| *Ara ararauna* | 1 | 5 | 80 | 38.5 - 99.9 |  |  |
| *Aratinga jandaya* | 1 | 2 | 50 | 0.9 - 99.2 |  |  |
| *Ardea cinerea* | 1 | 4 | 50 | 8.5 - 91.5 |  |  |
| *Asio otus* | 1 | 1 | 0 | 0 - 69 |  |  |
| *Athene noctua* | 1 | 6 | 0 | 0 - 15.2 |  |  |
| *Baeolophus bicolor* | 3 | 81 | 5.6 | 0 - 43.5 | 94.6 |  |
| *Basileuterus culicivorus* | 1 | 1 | 0 | 0 - 69 |  |  |
| *Basileuterus rufifrons* | 1 | 27 | 0 | 0 - 3.5 |  |  |
| *Bombycilla garrulus* | 1 | 10 | 10 | 0 - 34.9 |  |  |
| *Buteo buteo* | 1 | 1 | 0 | 0 - 69 |  |  |
| *Cardinalis cardinalis* | 3 | 83 | 0.5 | 0 - 4.1 | 25.5 |  |
| *Carduelis pinus* | 1 | 154 | 1.3 | 0.1 - 3.7 |  |  |
| *Catharus aurantiirostris* | 1 | 40 | 0 | 0 - 2.4 |  |  |
| *Catharus guttatus* | 1 | 3 | 0 | 0 - 28.7 |  |  |
| *Chiroxiphia linearis* | 1 | 59 | 0 | 0 - 1.6 |  |  |
| *Chloris chloris* | 1 | 2 | 0 | 0 - 40.8 |  |  |
| *Chlorostilbon aureoventris* | 1 | 1 | 0 | 0 - 69 |  |  |
| *Chrysomus ruficapillus* | 1 | 9 | 0 | 0 - 10.3 |  |  |
| *Coccothraustes vespertinus* | 2 | 3 | 100 | 71.3 - 100 | 0 |  |
| *Columba livia* | 1 | 28 | 0 | 0 - 3.4 |  |  |
| *Columba palumbus* | 2 | 82 | 1.2 | 0 - 4.7 | 0 |  |
| *Columbina talpacoti* | 1 | 25 | 0 | 0 - 3.8 |  |  |
| *Corvus brachyrhynchos* | 3 | 5 | 27 | 0 - 100 | 83.1 |  |
| *Corvus corone* | 2 | 41 | 0 | 0 - 2.3 | 0 |  |
| *Corvus frugilegus* | 1 | 13 | 30.8 | 9.7 - 57.4 |  |  |
| *Corvus monedula* | 2 | 14 | 0 | 0 - 6.7 | 0 |  |
| *Corvus splendens* | 1 | 94 | 4.3 | 1.1 - 9.2 |  |  |
| *Coturnix coturnix* | 1 | 1 | 0 | 0 - 69 |  |  |
| *Cyanocitta cristata* | 3 | 5 | 69.9 | 0 - 100 | 83.1 |  |
| *Cygnus olor* | 1 | 1 | 0 | 0 - 69 |  |  |
| *Dendroica coronata* | 1 | 27 | 0 | 0 - 3.5 |  |  |
| *Dumetella carolinensis* | 2 | 47 | 0 | 0 - 2 | 0 |  |
| *Elaenia flavogaster* | 1 | 2 | 0 | 0 - 40.8 |  |  |
| *Falco tinnunculus* | 1 | 2 | 0 | 0 - 40.8 |  |  |
| *Fluvicola nengeta* | 1 | 1 | 0 | 0 - 69 |  |  |
| *Fulica atra* | 1 | 2 | 0 | 0 - 40.8 |  |  |
| *Furnarius rufus* | 1 | 6 | 0 | 0 - 15.2 |  |  |
| *Garrulus glandarius* | 1 | 2 | 0 | 0 - 40.8 |  |  |
| *Geothlypis trichas* | 1 | 13 | 7.7 | 0 - 27.6 |  |  |
| *Guarouba guarouba* | 1 | 2 | 0 | 0 - 40.8 |  |  |
| *Haemorhous mexicanus* | 11 | 1384 | 53.5 | 20.8 - 84.6 | 99.3 |  |
| *Haemorhous purpureus* | 4 | 37 | 58 | 3.3 - 100 | 88.8 |  |
| *Icteria virens* | 1 | 2 | 0 | 0 - 40.8 |  |  |
| *Junco hyemalis* | 1 | 15 | 6.7 | 0 - 24.2 |  |  |
| *Larus argentatus* | 1 | 16 | 0 | 0 - 5.9 |  |  |
| *Meleagris gallopavo* | 1 | 1 | 0 | 0 - 69 |  |  |
| *Melospiza georgiana* | 1 | 1 | 0 | 0 - 69 |  |  |
| *Melospiza melodia* | 2 | 124 | 0.8 | 0 - 3.1 | 0 |  |
| *Melozone leucotis* | 1 | 43 | 0 | 0 - 2.2 |  |  |
| *Mimus polyglottos* | 2 | 12 | 0 | 0 - 7.8 | 0 |  |
| *Molothrus ater* | 3 | 21 | 0 | 0 - 4.5 | 0 |  |
| *Molothrus bonariensis* | 1 | 7 | 0 | 0 - 13.1 |  |  |
| *Momotus momota* | 1 | 17 | 0 | 0 - 5.5 |  |  |
| *Passer domesticus* | 5 | 605 | 0 | 0 - 0.3 | 0 |  |
| *Pavo muticus* | 1 | 1 | 0 | 0 - 69 |  |  |
| *Perdix perdix* | 1 | 6 | 0 | 0 - 15.2 |  |  |
| *Phasianus colchicus* | 1 | 7 | 0 | 0 - 13.1 |  |  |
| *Pica pica* | 2 | 13 | 6 | 0 - 24.6 | 0 |  |
| *Picoides pubescens* | 1 | 36 | 2.8 | 0 - 10.6 |  |  |
| *Pinicola enucleator* | 1 | 3 | 100 | 71.3 - 100 |  |  |
| *Pionus fuscus* | 1 | 3 | 66.7 | 14.4 - 99.8 |  |  |
| *Pipilo erythrophthalmus* | 1 | 7 | 0 | 0 - 13.1 |  |  |
| *Pitangus sulphuratus* | 1 | 2 | 0 | 0 - 40.8 |  |  |
| *Poecile atricapillus* | 3 | 163 | 14.5 | 0 - 82.2 | 79.6 |  |
| *Poecile carolinensis* | 2 | 18 | 0 | 0 - 5.2 | 0 |  |
| *Quiscalus quiscula* | 1 | 3 | 0 | 0 - 28.7 |  |  |
| *Ramphodon naevius* | 1 | 1 | 0 | 0 - 69 |  |  |
| *Regulus calendula* | 1 | 9 | 0 | 0 - 10.3 |  |  |
| *Regulus satrapa* | 1 | 5 | 0 | 0 - 18 |  |  |
| *Setophaga coronata* | 1 | 1 | 0 | 0 - 69 |  |  |
| *Sicalis flaveola* | 1 | 37 | 0 | 0 - 2.6 |  |  |
| *Sitta carolinensis* | 1 | 19 | 0 | 0 - 5 |  |  |
| *Spinus psaltria* | 2 | 4 | 100 | 77.9 - 100 | 0 |  |
| *Spinus spinus* | 1 | 1 | 0 | 0 - 69 |  |  |
| *Spinus tristis* | 6 | 643 | 29.3 | 7.7 - 57.9 | 94 |  |
| *Spizella arborea* | 2 | 48 | 2 | 0 - 7.8 | 0 |  |
| *Spizella passerina* | 2 | 21 | 0 | 0 - 4.5 | 0 |  |
| *Streptopelia decaocto* | 1 | 1 | 0 | 0 - 69 |  |  |
| *Strix aluco* | 1 | 13 | 0 | 0 - 7.2 |  |  |
| *Sturnus vulgaris* | 3 | 7 | 0 | 0 - 13.1 | 0 |  |
| *Tachyphonus coronatus* | 1 | 1 | 0 | 0 - 69 |  |  |
| *Thraupis sayaca* | 1 | 1 | 0 | 0 - 69 |  |  |
| *Thryophilus rufalbus* | 1 | 21 | 0 | 0 - 4.5 |  |  |
| *Thryothorus ludovicianus* | 1 | 6 | 0 | 0 - 15.2 |  |  |
| *Todirostrum cinereum* | 1 | 2 | 0 | 0 - 40.8 |  |  |
| *Toxostoma rufum* | 1 | 9 | 0 | 0 - 10.3 |  |  |
| *Troglodytes aedon* | 2 | 2 | 0 | 0 - 40.8 | 0 |  |
| *Turdus amaurochalinus* | 1 | 4 | 0 | 0 - 22.2 |  |  |
| *Turdus flavipes* | 1 | 1 | 0 | 0 - 69 |  |  |
| *Turdus merula* | 1 | 3 | 0 | 0 - 28.7 |  |  |
| *Turdus migratorius* | 4 | 30 | 0 | 0 - 3.2 | 0 |  |
| *Turdus rufivenaris* | 1 | 1 | 0 | 0 - 69 |  |  |
| *Tyto alba* | 1 | 7 | 0 | 0 - 13.1 |  |  |
| *Zenaida macroura* | 1 | 54 | 5.6 | 1.1 - 13.2 |  |  |
| *Zonotrichia albicollis* | 3 | 51 | 0.9 | 0 - 6.1 | 16.4 |  |
| *Zonotrichia capensis* | 1 | 8 | 0 | 0 - 11.5 |  |  |
| *Zonotrichia leucophrys* | 1 | 23 | 4.4 | 0 - 16.2 |  |  |
| **Order** | | | | | | p<0.0001 |
| *Accipitriformes* | 1 | 1 | 0 | 0 - 69 |  |  |
| *Anseriformes* | 1 | 62 | 1.6 | 0 - 6.2 |  |  |
| *Apodiformes* | 1 | 2 | 0 | 0 - 40.8 |  |  |
| *Charadriiformes* | 1 | 16 | 0 | 0 - 5.9 |  |  |
| *Columbiformes* | 4 | 190 | 1.5 | 0 - 5 | 36.7 |  |
| *Coraciiformes* | 1 | 17 | 0 | 0 - 5.5 |  |  |
| *Falconiformes* | 1 | 2 | 0 | 0 - 40.8 |  |  |
| *Galliformes* | 2 | 16 | 0 | 0 - 5.9 | 0 |  |
| *Gruiformes* | 1 | 2 | 0 | 0 - 40.8 |  |  |
| *Passeriformes* | 19 | 4237 | 27.3 | 12 - 46.1 | 99.3 |  |
| *Pelecaniformes* | 1 | 4 | 50 | 8.5 - 91.5 |  |  |
| *Piciformes* | 1 | 36 | 2.8 | 0 - 10.6 |  |  |
| *Psittaciformes* | 1 | 75 | 52 | 40.7 - 63.2 |  |  |
| *Strigiformes* | 1 | 27 | 0 | 0 - 3.5 |  |  |
| **Wild vs. captive** | | | | | | p=0.024 |
| Captive | 1 | 75 | 52 | 40.7 - 63.2 |  |  |
| Unknown | 11 | 2454 | 26.9 | 12.9 - 43.8 | 99.7 |  |
| Wild | 9 | 2158 | 22.4 | 1.9 - 56.3 | 99.6 |  |
