# Supplementary material for: Occurrence of Mycoplasma gallisepticum in wild birds: A systematic review and meta-analysis
Source: PLoS One. 2020 Apr 16;15(4):e0231545. doi: 10.1371/journal.pone.0231545 (PMC7162529; doi:10.1371/journal.pone.0231545)
Supplement: S7 Table — (DOCX) [file pone.0231545.s008.docx]

S8 Table. Subgroup meta-analysis of the MG prevalence by SPA.

| **Subgroup** | **No of prevalence inputs** | **Sample size** | **Mean (%)** | **95% CI** | **I^2^ (%)** | **Difference between groups** |
| --- | --- | --- | --- | --- | --- | --- |
| **Country** | | | | | | p<0.0001 |
| Brazil | 2 | 194 | 2.3 | 0.2 - 6.5 | 54.1 |  |
| Malaysia | 1 | 45 | 0 | 0 - 2.1 |  |  |
| Mexico | 1 | 251 | 0 | 0 - 0.4 |  |  |
| Namibia and South Africa | 1 | 234 | 8.1 | 5 - 12 |  |  |
| USA | 26 | 8399 | 15.5 | 9 - 23.4 | 98.7 |  |
| **Region** | | | | | | p<0.0001 |
| Africa | 1 | 234 | 8.1 | 5 - 12 |  |  |
| Asia | 1 | 45 | 0 | 0 - 2.1 |  |  |
| North America | 27 | 8650 | 14.3 | 8.2 - 21.9 | 98.7 |  |
| South America | 2 | 194 | 2.3 | 0.2 - 6.5 | 54.1 |  |
| **Species** | | | | | | p<0.0001 |
| *Agelaius phoeniceus* | 2 | 75 | 42.2 | 0 - 100 | 89.7 |  |
| *Anas diazi* | 1 | 26 | 0 | 0 - 3.7 |  |  |
| *Aquila chrysaetos* | 1 | 26 | 0 | 0 - 3.7 |  |  |
| *Asio clamator* | 1 | 11 | 0 | 0 - 8.5 |  |  |
| *Asio stygius* | 1 | 3 | 0 | 0 - 28.7 |  |  |
| *Athene cunicularia* | 1 | 1 | 0 | 0 - 69 |  |  |
| *Baeolophus bicolor* | 4 | 89 | 52.7 | 3.7 - 98.1 | 96.9 |  |
| *Bombycilla garrulus* | 1 | 10 | 0 | 0 - 9.3 |  |  |
| *Bubo virginianus* | 1 | 1 | 0 | 0 - 69 |  |  |
| *Bubulcus ibis* | 1 | 17 | 0 | 0 - 5.5 |  |  |
| *Buteo albicaudatus* | 1 | 1 | 0 | 0 - 69 |  |  |
| *Buteo brachyurus* | 1 | 2 | 0 | 0 - 40.8 |  |  |
| *Callipepla squamata* | 1 | 6 | 0 | 0 - 15.2 |  |  |
| *Caracara plancus* | 1 | 12 | 0 | 0 - 7.8 |  |  |
| *Cardinalis cardinalis* | 3 | 83 | 41.3 | 3.6 - 87.2 | 94.2 |  |
| *Carduelis pinus* | 1 | 154 | 2 | 0.4 - 4.7 |  |  |
| *Cathartes aura* | 2 | 57 | 0 | 0 - 1.7 | 0 |  |
| *Catharus guttatus* | 1 | 3 | 0 | 0 - 28.7 |  |  |
| *Charadrius vociferus* | 1 | 1 | 0 | 0 - 69 |  |  |
| *Coccothraustes vespertinus* | 1 | 8 | 0 | 0 - 11.5 |  |  |
| *Colinus virginianus* | 3 | 38 | 0 | 0 - 2.5 | 0 |  |
| *Columbina inca* | 1 | 1 | 0 | 0 - 69 |  |  |
| *Coragyps atratus* | 1 | 9 | 11.1 | 0 - 38.2 |  |  |
| *Corvus splendens* | 1 | 45 | 0 | 0 - 2.1 |  |  |
| *Crotophaga sulcirostris* | 1 | 3 | 0 | 0 - 28.7 |  |  |
| *Crypturellus obsoletus* | 1 | 3 | 0 | 0 - 28.7 |  |  |
| *Crypturellus parvirostris* | 1 | 20 | 0 | 0 - 4.7 |  |  |
| *Crypturellus tataupa* | 1 | 2 | 0 | 0 - 40.8 |  |  |
| *Crypturellus undulatus* | 1 | 10 | 0 | 0 - 9.3 |  |  |
| *Cyanocitta cristata* | 1 | 3 | 33.3 | 0.3 - 85.6 |  |  |
| *Dendrocygna autumnalis* | 1 | 153 | 0 | 0 - 0.6 |  |  |
| *Dendrocygna bicolor* | 1 | 1 | 0 | 0 - 69 |  |  |
| *Dendroica coronata* | 1 | 27 | 0 | 0 - 3.5 |  |  |
| *Dumetella carolinensis* | 2 | 47 | 58.5 | 0 - 100 | 92.4 |  |
| *Egretta tricolor* | 1 | 2 | 0 | 0 - 40.8 |  |  |
| *Falco femoralis* | 1 | 2 | 0 | 0 - 40.8 |  |  |
| *Falco rufigularis* | 1 | 1 | 0 | 0 - 69 |  |  |
| *Falco sparverius* | 1 | 2 | 0 | 0 - 40.8 |  |  |
| *Fulica americana* | 1 | 1 | 0 | 0 - 69 |  |  |
| *Geothlypis trichas* | 1 | 13 | 0 | 0 - 7.2 |  |  |
| *Glaucidium brasilianum* | 1 | 6 | 0 | 0 - 15.2 |  |  |
| *Gymnogyps californianus* | 1 | 120 | 14.2 | 8.5 - 20.9 |  |  |
| *Haemorhous mexicanus* | 8 | 1356 | 28.4 | 11.9 - 48.7 | 98.1 |  |
| *Haemorhous purpureus* | 2 | 52 | 3.2 | 0 - 25.8 | 85.2 |  |
| *Heterospizias meridionalis* | 1 | 1 | 0 | 0 - 69 |  |  |
| *Icteria virens* | 2 | 4 | 0 | 0 - 22.2 | 0 |  |
| *Junco hyemalis* | 2 | 20 | 0 | 0 - 4.7 | 0 |  |
| *Leptodon cayanensis* | 1 | 2 | 0 | 0 - 40.8 |  |  |
| *Megascops choliba* | 1 | 6 | 0 | 0 - 15.2 |  |  |
| *Meleagris gallopavo* | 14 | 3883 | 10.7 | 3.3 - 21.6 | 98.5 |  |
| *Melospiza georgiana* | 1 | 1 | 0 | 0 - 69 |  |  |
| *Melospiza melodia* | 3 | 125 | 5.4 | 2.2 - 10.1 | 0 |  |
| *Milvago chimachima* | 1 | 5 | 0 | 0 - 18 |  |  |
| *Mimus polyglottos* | 2 | 12 | 66.1 | 0 - 100 | 73.9 |  |
| *Molothrus aeneus* | 1 | 1 | 0 | 0 - 69 |  |  |
| *Molothrus ater* | 6 | 205 | 20 | 3.1 - 46.6 | 80.7 |  |
| *Passer domesticus* | 3 | 459 | 6.2 | 1.4 - 14.1 | 82.2 |  |
| *Picoides pubescens* | 1 | 36 | 2.8 | 0 - 10.6 |  |  |
| *Pipilo erythrophthalmus* | 1 | 7 | 0 | 0 - 13.1 |  |  |
| *Poecile atricapillus* | 1 | 160 | 6.9 | 3.5 - 11.3 |  |  |
| *Poecile carolinensis* | 2 | 18 | 57.8 | 0 - 100 | 82.2 |  |
| *Psilorhinus morio* | 1 | 5 | 0 | 0 - 18 |  |  |
| *Quiscalus mexicanus* | 1 | 3 | 0 | 0 - 28.7 |  |  |
| *Quiscalus quiscula* | 2 | 143 | 84.7 | 23.7 - 100 | 81.8 |  |
| *Regulus calendula* | 1 | 9 | 0 | 0 - 10.3 |  |  |
| *Regulus satrapa* | 1 | 5 | 0 | 0 - 18 |  |  |
| *Rhynchotus rufescens* | 1 | 40 | 7.5 | 1.5 - 17.6 |  |  |
| *Rupornis magnirostris* | 1 | 5 | 0 | 0 - 18 |  |  |
| *Setophaga coronata* | 1 | 1 | 100 | 31 - 100 |  |  |
| *Setophaga pinus* | 1 | 4 | 0 | 0 - 22.2 |  |  |
| *Sitta carolinensis* | 1 | 19 | 0 | 0 - 5 |  |  |
| *Spheniscus demersus* | 1 | 234 | 8.1 | 5 - 12 |  |  |
| *Spinus tristis* | 4 | 590 | 21.6 | 2.6 - 52 | 91.8 |  |
| *Spizella arborea* | 2 | 61 | 1.7 | 0 - 10.6 | 49.9 |  |
| *Spizella passerina* | 2 | 21 | 62.3 | 0 - 100 | 78.6 |  |
| *Streptopelia decaocto* | 1 | 1 | 0 | 0 - 69 |  |  |
| *Strix huhula* | 1 | 1 | 0 | 0 - 69 |  |  |
| *Strix virgata* | 1 | 1 | 0 | 0 - 69 |  |  |
| *Sturnella magna* | 1 | 24 | 16.7 | 4.8 - 33.8 |  |  |
| *Sturnus vulgaris* | 2 | 98 | 42.2 | 0 - 100 | 89.8 |  |
| *Thryothorus ludovicianus* | 1 | 6 | 0 | 0 - 15.2 |  |  |
| *Tinamus solitarius* | 1 | 20 | 5 | 0 - 18.5 |  |  |
| *Toxostoma rufum* | 2 | 10 | 24.4 | 0 - 84.6 | 47.8 |  |
| *Troglodytes aedon* | 1 | 1 | 0 | 0 - 69 |  |  |
| *Turdus migratorius* | 3 | 24 | 37.9 | 0 - 98.8 | 87.8 |  |
| *Tympanuchius pallidicinctus* | 1 | 162 | 4.9 | 2.2 - 8.8 |  |  |
| *Tyto alba* | 2 | 30 | 0 | 0 - 3.2 | 0 |  |
| *Zenaida asiatica* | 1 | 11 | 0 | 0 - 8.5 |  |  |
| *Zenaida macroura* | 2 | 62 | 0 | 0 - 13.8 | 0 |  |
| *Zonotrichia albicollis* | 4 | 61 | 12.6 | 0 - 49.8 | 89.3 |  |
| *Zonotrichia leucophrys* | 1 | 23 | 4.4 | 0 - 16.2 |  |  |
| **Order** | | | | | | p<0.0001 |
| *Accipitriformes* | 3 | 223 | 8 | 4.8 - 11.9 | 0 |  |
| *Anseriformes* | 1 | 180 | 0 | 0 - 0.5 |  |  |
| *Charadriiformes* | 1 | 1 | 0 | 0 - 69 |  |  |
| *Columbiformes* | 3 | 75 | 0 | 0 - 12.8 | 0 |  |
| *Cuculiformes* | 1 | 3 | 0 | 0 - 28.7 |  |  |
| *Falconiformes* | 1 | 22 | 0 | 0 - 4.3 |  |  |
| *Galliformes* | 17 | 4089 | 7.8 | 2.3 - 16.1 | 98.2 |  |
| *Gruiformes* | 1 | 1 | 0 | 0 - 69 |  |  |
| *Passeriformes* | 12 | 4085 | 19.9 | 9.5 - 32.9 | 98.6 |  |
| *Pelecaniformes* | 1 | 19 | 0 | 0 - 5 |  |  |
| *Piciformes* | 1 | 36 | 2.8 | 0 - 10.6 |  |  |
| *Sphenisciformes* | 1 | 234 | 8.1 | 5 - 12 |  |  |
| *Strigiformes* | 2 | 60 | 0 | 0 - 1.6 | 0 |  |
| *Tinamiformes* | 1 | 95 | 4.2 | 1.1 - 9.1 |  |  |
| **Wild versus captive** | | | | | | p=0.8516 |
| captive | 2 | 123 | 25.3 | 0 - 84.4 | 97.3 |  |
| unknown | 7 | 3327 | 10.9 | 1 - 29.8 | 99.3 |  |
| wild | 24 | 5673 | 11.3 | 5.7 - 18.3 | 98 |  |
