# Supplementary material for: Occurrence of Mycoplasma gallisepticum in wild birds: A systematic review and meta-analysis
Source: PLoS One. 2020 Apr 16;15(4):e0231545. doi: 10.1371/journal.pone.0231545 (PMC7162529; doi:10.1371/journal.pone.0231545)
Supplement: S8 Table — (DOCX) [file pone.0231545.s009.docx]

S9 Table. Subgroup meta-analysis of the MG prevalence by HI.

| **Subgroup** | **No of prevalence inputs** | **Sample size** | **Mean (%)** | **95% CI** | **I^2^ (%)** | **Difference between groups** |
| --- | --- | --- | --- | --- | --- | --- |
| **Country** | | | | | | p<0.0001 |
| Brazil | 2 | 96 | 0 | 0 - 1 | 0 |  |
| Costa Rica | 1 | 241 | 0 | 0 - 0.4 |  |  |
| Galápagos Islands | 1 | 250 | 0 | 0 - 0.4 |  |  |
| USA | 17 | 4775 | 6.6 | 2.7 - 12.2 | 97.2 |  |
| **Region** | | | | | | p<0.0001 |
| North America | 18 | 5016 | 5.8 | 2.3 - 10.8 | 97.1 |  |
| South America | 3 | 346 | 0 | 0 - 0.3 |  |  |
| **Species** | | | | | | p<0.0001 |
| *Atlapetes albinucha* | 1 | 1 | 0 | 0 - 69 |  |  |
| *Aulacorhynchus caeruleogularis* | 1 | 4 | 0 | 0 - 22.2 |  |  |
| *Baeolophus bicolor* | 1 | 25 | 28 | 12.5 - 46.8 |  |  |
| *Camarhynchus crassirostris* | 1 | 5 | 0 | 0 - 18 |  |  |
| *Camarhynchus pallidus* | 1 | 2 | 0 | 0 - 40.8 |  |  |
| *Camarhynchus parvulus* | 1 | 14 | 0 | 0 - 6.7 |  |  |
| *Cardinalis cardinalis* | 1 | 6 | 0 | 0 - 15.2 |  |  |
| *Catharus aurantiirostris* | 1 | 29 | 0 | 0 - 3.3 |  |  |
| *Catharus ustulatus* | 1 | 10 | 0 | 0 - 9.3 |  |  |
| *Certhidea olivacea* | 1 | 1 | 0 | 0 - 69 |  |  |
| *Chiroxiphia linearis* | 1 | 2 | 0 | 0 - 40.8 |  |  |
| *Colinus virginianus* | 1 | 7 | 0 | 0 - 13.1 |  |  |
| *Coragyps atratus* | 1 | 1 | 0 | 0 - 69 |  |  |
| *Crotophaga ani* | 1 | 2 | 0 | 0 - 40.8 |  |  |
| *Crypturellus obsoletus* | 1 | 3 | 0 | 0 - 28.7 |  |  |
| *Crypturellus parvirostris* | 1 | 20 | 0 | 0 - 4.7 |  |  |
| *Crypturellus tataupa* | 1 | 2 | 0 | 0 - 40.8 |  |  |
| *Crypturellus undulatus* | 1 | 10 | 0 | 0 - 9.3 |  |  |
| *Dendrocincla homochroa* | 1 | 20 | 0 | 0 - 4.7 |  |  |
| *Dendrocolaptes sanctithomae* | 1 | 2 | 0 | 0 - 40.8 |  |  |
| *Dendroica petechia* | 1 | 26 | 0 | 0 - 3.7 |  |  |
| *Euphonia hirundinacea* | 1 | 1 | 0 | 0 - 69 |  |  |
| *Falco mexicanus* | 1 | 34 | 2.9 | 0 - 11.2 |  |  |
| *Geospiza fortis* | 1 | 56 | 0 | 0 - 1.7 |  |  |
| *Geospiza fuliginosa* | 1 | 137 | 0 | 0 - 0.7 |  |  |
| *Gymnogyps californianus* | 1 | 17 | 100 | 94.5 - 100 |  |  |
| *Habia rubica* | 1 | 4 | 0 | 0 - 22.2 |  |  |
| *Haemorhous mexicanus* | 4 | 729 | 12.7 | 0.4 - 38 | 97.5 |  |
| *Haemorhous purpureus* | 1 | 9 | 0 | 0 - 10.3 |  |  |
| *Hylocichla mustelina* | 1 | 5 | 0 | 0 - 18 |  |  |
| *Icterus galbula* | 1 | 1 | 0 | 0 - 69 |  |  |
| *Lepidocolaptes souleyetii* | 1 | 2 | 0 | 0 - 40.8 |  |  |
| *Leptotila verreauxi* | 1 | 7 | 0 | 0 - 13.1 |  |  |
| *Megarynchus pitangua* | 1 | 1 | 0 | 0 - 69 |  |  |
| *Meleagris gallopavo* | 10 | 1909 | 1.6 | 0.1 - 4.8 | 92.7 |  |
| *Melospiza melodia* | 1 | 31 | 0 | 0 - 3.1 |  |  |
| *Melozone leucotis* | 1 | 58 | 0 | 0 - 1.7 |  |  |
| *Mimus polyglottos* | 1 | 1 | 0 | 0 - 69 |  |  |
| *Molothrus ater* | 2 | 167 | 0.6 | 0 - 2.3 | 0 |  |
| *Momotus momota* | 1 | 15 | 0 | 0 - 6.3 |  |  |
| *Myiarchus magnirostris* | 1 | 1 | 0 | 0 - 69 |  |  |
| *Nesomimus parvulus* | 1 | 6 | 0 | 0 - 15.2 |  |  |
| *Passer domesticus* | 2 | 682 | 0.2 | 0 - 2.1 | 84.8 |  |
| *Pitangus sulphuratus* | 1 | 1 | 0 | 0 - 69 |  |  |
| *Psilorhinus morio* | 1 | 1 | 0 | 0 - 69 |  |  |
| *Quiscalus quiscula* | 2 | 136 | 14.6 | 0 - 73.2 | 80.4 |  |
| *Rhynchotus rufescens* | 1 | 40 | 0 | 0 - 2.4 |  |  |
| *Saltator coerulescens* | 1 | 1 | 0 | 0 - 69 |  |  |
| *Saltator maximus* | 1 | 3 | 0 | 0 - 28.7 |  |  |
| *Setophaga coronata* | 1 | 1 | 0 | 0 - 69 |  |  |
| *Setophaga pinus* | 1 | 4 | 0 | 0 - 22.2 |  |  |
| *Spinus tristis* | 1 | 11 | 0 | 0 - 8.5 |  |  |
| *Sturnella magna* | 1 | 24 | 16.7 | 4.8 - 33.8 |  |  |
| *Sturnus vulgaris* | 3 | 966 | 0.1 | 0 - 1.3 | 44.6 |  |
| *Thraupis episcopus* | 1 | 2 | 0 | 0 - 40.8 |  |  |
| *Thryophilus rufalbus* | 1 | 24 | 0 | 0 - 4 |  |  |
| *Tinamus solitarius* | 1 | 20 | 0 | 0 - 4.7 |  |  |
| *Toxostoma rufum* | 1 | 1 | 0 | 0 - 69 |  |  |
| *Turdus assimilis* | 1 | 14 | 0 | 0 - 6.7 |  |  |
| *Turdus grayi* | 1 | 33 | 0 | 0 - 2.9 |  |  |
| *Turdus migratorius* | 2 | 4 | 0 | 0 - 22.2 | 0 |  |
| *Zenaida macroura* | 1 | 8 | 0 | 0 - 11.5 |  |  |
| *Zonotrichia albicollis* | 1 | 3 | 0 | 0 - 28.7 |  |  |
| **Order** | | | | | | p=0.3145 |
| *Accipitriformes* | 2 | 18 | 57.5 | 0 - 100 | 89.3 |  |
| *Columbiformes* | 2 | 15 | 0 | 0 - 6.3 | 0 |  |
| *Coraciiformes* | 1 | 15 | 0 | 0 - 6.3 |  |  |
| *Cuculiformes* | 1 | 2 | 0 | 0 - 40.8 |  |  |
| *Falconiformes* | 1 | 34 | 2.9 | 0 - 11.2 |  |  |
| *Galliformes* | 11 | 1916 | 1.4 | 0.1 - 4.4 | 91.9 |  |
| *Passeriformes* | 7 | 3263 | 4.5 | 0.5 - 12.3 | 98.1 |  |
| *Piciformes* | 1 | 4 | 0 | 0 - 22.2 |  |  |
| *Tinamiformes* | 1 | 95 | 0 | 0 - 1 |  |  |
| **Wild versus captive** | | | | | | p=0.5573 |
| captive | 2 | 111 | 49.6 | 0 - 100 | 99.3 |  |
| unknown | 3 | 599 | 7 | 0 - 29 | 97.9 |  |
| wild | 17 | 4652 | 2.3 | 0.5 - 5.3 | 95.3 |  |
