# Supplementary material for: Occurrence of Mycoplasma gallisepticum in wild birds: A systematic review and meta-analysis
Source: PLoS One. 2020 Apr 16;15(4):e0231545. doi: 10.1371/journal.pone.0231545 (PMC7162529; doi:10.1371/journal.pone.0231545)
Supplement: S9 Table — (DOCX) [file pone.0231545.s010.docx]

S10 Table. Subgroup meta-analysis of the MG prevalence by ELISA.

| **Subgroup** | **No of prevalence inputs** | **Sample size** | **Mean (%)** | **95% CI** | **I^2^ (%)** | **Difference between groups** |
| --- | --- | --- | --- | --- | --- | --- |
| **Country** |  |  |  |  |  | p<0.0001 |
| Belgium | 1 | 698 | 0.7 | 0.2 - 1.5 |  |  |
| Galápagos Islands | 1 | 88 | 0 | 0 - 1.1 |  |  |
| Malaysia | 1 | 45 | 60 | 45.5 - 73.7 |  |  |
| Namibia and South Africa | 1 | 189 | 0.5 | 0 - 2.1 |  |  |
| USA | 2 | 138 | 7.2 | 0 - 52 | 97.4 |  |
| **Region** |  |  |  |  |  | p<0.0001 |
| Africa | 1 | 189 | 0.5 | 0 - 2.1 |  |  |
| Asia | 1 | 45 | 60 | 45.5 - 73.7 |  |  |
| Europe | 1 | 698 | 0.7 | 0.2 - 1.5 |  |  |
| North America | 2 | 138 | 7.2 | 0 - 52 | 97.4 |  |
| South America | 1 | 88 | 0 | 0 - 1.1 |  |  |
| **Species** | | | | | | p<0.0001 |
| *Anas platyrhynchos* | 1 | 57 | 1.8 | 0 - 6.7 |  |  |
| *Anser anser* | 1 | 4 | 0 | 0 - 22.2 |  |  |
| *Ardea cinerea* | 1 | 4 | 50 | 8.5 - 91.5 |  |  |
| *Asio otus* | 1 | 1 | 0 | 0 - 69 |  |  |
| *Athene noctua* | 1 | 6 | 0 | 0 - 15.2 |  |  |
| *Buteo buteo* | 1 | 1 | 0 | 0 - 69 |  |  |
| *Columba livia* | 1 | 28 | 0 | 0 - 3.4 |  |  |
| *Columba palumbus* | 1 | 80 | 1.3 | 0 - 4.8 |  |  |
| *Corvus corone* | 1 | 35 | 0 | 0 - 2.7 |  |  |
| *Corvus monedula* | 1 | 13 | 0 | 0 - 7.2 |  |  |
| *Corvus splendens* | 1 | 45 | 60 | 45.5 - 73.7 |  |  |
| *Coturnix coturnix* | 1 | 1 | 0 | 0 - 69 |  |  |
| *Cygnus olor* | 1 | 1 | 0 | 0 - 69 |  |  |
| *Falco tinnunculus* | 1 | 2 | 0 | 0 - 40.8 |  |  |
| *Fulica atra* | 1 | 2 | 0 | 0 - 40.8 |  |  |
| *Garrulus glandarius* | 1 | 2 | 0 | 0 - 40.8 |  |  |
| *Haemorhous mexicanus* | 1 | 52 | 26.9 | 15.9 - 39.7 |  |  |
| *Larus argentatus* | 1 | 16 | 0 | 0 - 5.9 |  |  |
| *Meleagris gallopavo* | 1 | 86 | 0 | 0 - 1.1 |  |  |
| *Mimus trifasciatus* | 1 | 88 | 0 | 0 - 1.1 |  |  |
| *Passer domesticus* | 1 | 401 | 0 | 0 - 0.2 |  |  |
| *Pavo muticus* | 1 | 1 | 0 | 0 - 69 |  |  |
| *Perdix perdix* | 1 | 6 | 0 | 0 - 15.2 |  |  |
| *Phasianus colchicus* | 1 | 7 | 0 | 0 - 13.1 |  |  |
| *Pica pica* | 1 | 10 | 10 | 0 - 34.9 |  |  |
| *Spheniscus demersus* | 1 | 189 | 0.5 | 0 - 2.1 |  |  |
| *Strix aluco* | 1 | 13 | 0 | 0 - 7.2 |  |  |
| *Tyto alba* | 1 | 7 | 0 | 0 - 13.1 |  |  |
| **Order** | | | | | | p=0.1385 |
| *Accipitriformes* | 1 | 1 | 0 | 0 - 69 |  |  |
| *Anseriformes* | 1 | 62 | 1.6 | 0 - 6.2 |  |  |
| *Charadriiformes* | 1 | 16 | 0 | 0 - 5.9 |  |  |
| *Columbiformes* | 1 | 108 | 0.9 | 0 - 3.6 |  |  |
| *Falconiformes* | 1 | 2 | 0 | 0 - 40.8 |  |  |
| *Galliformes* | 2 | 101 | 0 | 0 - 1 | 0 |  |
| *Gruiformes* | 1 | 2 | 0 | 0 - 40.8 |  |  |
| *Passeriformes* | 4 | 646 | 12.7 | 0 - 43.7 | 98.1 |  |
| *Pelecaniformes* | 1 | 4 | 50 | 8.5 - 91.5 |  |  |
| *Sphenisciformes* | 1 | 189 | 0.5 | 0 - 2.1 |  |  |
| *Strigiformes* | 1 | 27 | 0 | 0 - 3.5 |  |  |
| **Wild versus captive** | | | | | | p<0.0001 |
| Unknown |  | 97 | 43 | 13.8 - 75.1 | 91.1 |  |
| Wild |  | 1061 | 0.4 | 0 - 1 | 25.3 |  |

sss
