# Supplementary material for: Occurrence of Mycoplasma gallisepticum in wild birds: A systematic review and meta-analysis
Source: PLoS One. 2020 Apr 16;15(4):e0231545. doi: 10.1371/journal.pone.0231545 (PMC7162529; doi:10.1371/journal.pone.0231545)
Supplement: S10 Table — (DOCX) [file pone.0231545.s011.docx]

S11 Table. Subgroup meta-analysis of the MG prevalence by other methods.

| **Subgroup** | **No of prevalence inputs** | **Sample size** | **Mean (%)** | **95% CI** | **I^2^ (%)** | **Difference between groups** |
| --- | --- | --- | --- | --- | --- | --- |
| **Country** |  |  |  |  |  | p=0.1215 |
| Germany | 1 | 40 | 0 | 0 - 2.4 |  |  |
| Japan | 1 | 217 | 1.8 | 0.5 - 4.1 |  |  |
| Spain | 1 | 17 | 11.8 | 1.3 - 30.8 |  |  |
| United Arab Emirates | 1 | 20 | 0 | 0 - 4.7 |  |  |
| USA | 2 | 346 | 1.3 | 0 - 10.5 | 92.6 |  |
| **Region** |  |  |  |  |  | p=0.9585 |
| Asia | 2 | 217 | 1.2 | 0 - 4.6 | 26.4 |  |
| Europe | 2 | 20 | 2.6 | 0 - 23.4 | 82.9 |  |
| North America | 2 | 346 | 1.3 | 0 - 10.5 | 92.6 |  |
| **Species** |  |  |  |  |  | p=1 |
| *Accipiter gentilis* | 1 | 13 | 0 | 0 - 7.2 |  |  |
| *Anas clypeata* | 2 | 4 | 0 | 0 - 22.2 | 0 |  |
| *Anas crecca* | 1 | 3 | 0 | 0 - 28.7 |  |  |
| *Anas falcata* | 1 | 2 | 0 | 0 - 40.8 |  |  |
| *Anas platyrhynchos* | 1 | 17 | 0 | 0 - 5.5 |  |  |
| *Anthropoides virgo* | 1 | 1 | 0 | 0 - 69 |  |  |
| *Ara macao* | 1 | 4 | 0 | 0 - 22.2 |  |  |
| *Ardea purpurea* | 1 | 2 | 0 | 0 - 40.8 |  |  |
| *Balearica pavonina* | 1 | 1 | 0 | 0 - 69 |  |  |
| *Bambusicola thoracica* | 1 | 8 | 0 | 0 - 11.5 |  |  |
| *Buteo buteo* | 1 | 7 | 0 | 0 - 13.1 |  |  |
| *Circus aeruginosus* | 1 | 8 | 0 | 0 - 11.5 |  |  |
| *Corvus macrorhynchos* | 1 | 1 | 0 | 0 - 69 |  |  |
| *Cygnus bewickii* | 1 | 5 | 0 | 0 - 18 |  |  |
| *Egretta garzetta* | 1 | 1 | 0 | 0 - 69 |  |  |
| *Falco biarmicus* | 1 | 2 | 0 | 0 - 40.8 |  |  |
| *Falco cherrug* | 1 | 9 | 0 | 0 - 10.3 |  |  |
| *Falco peregrinoides* | 1 | 2 | 0 | 0 - 40.8 |  |  |
| *Falco peregrinus* | 2 | 7 | 47.6 | 0 - 100 | 92.9 |  |
| *Falco rusticolus* | 1 | 2 | 0 | 0 - 40.8 |  |  |
| *Falco subbuteo* | 1 | 1 | 0 | 0 - 69 |  |  |
| *Falco tinnunculus* | 1 | 5 | 0 | 0 - 18 |  |  |
| *Fulica atra* | 2 | 5 | 0 | 0 - 18 | 0 |  |
| *Gallicrex cinerea* | 1 | 1 | 0 | 0 - 69 |  |  |
| *Gallinula chloropus* | 1 | 3 | 0 | 0 - 28.7 |  |  |
| *Gracula religiosa* | 1 | 3 | 0 | 0 - 28.7 |  |  |
| *Gyps fulvus* | 1 | 4 | 0 | 0 - 22.2 |  |  |
| *Hypsipetes amaurotis* | 1 | 1 | 0 | 0 - 69 |  |  |
| *Larvivora cyane* | 1 | 5 | 0 | 0 - 18 |  |  |
| *Leptoptilos dubius* | 1 | 2 | 0 | 0 - 40.8 |  |  |
| *Lonchura striata var. domestica* | 1 | 3 | 0 | 0 - 28.7 |  |  |
| *Meleagris gallopavo* | 2 | 346 | 1.3 | 0 - 10.5 | 92.6 |  |
| *Melopsittacus sp.* | 1 | 2 | 0 | 0 - 40.8 |  |  |
| *Milvus migrans* | 1 | 5 | 0 | 0 - 18 |  |  |
| *Nycticorax nycticorax* | 1 | 9 | 0 | 0 - 10.3 |  |  |
| *Passer domesticus* | 1 | 3 | 0 | 0 - 28.7 |  |  |
| *Passer montanus* | 1 | 94 | 4.3 | 1.1 - 9.2 |  |  |
| *Pavo cristatus* | 1 | 1 | 0 | 0 - 69 |  |  |
| *Phasianus colchicus* | 1 | 21 | 0 | 0 - 4.5 |  |  |
| *Phasianus soemmerringii* | 1 | 4 | 0 | 0 - 22.2 |  |  |
| *Phoenicopterus roseus* | 1 | 2 | 0 | 0 - 40.8 |  |  |
| *Picus awokera* | 1 | 1 | 0 | 0 - 69 |  |  |
| *Psittacula sp.* | 1 | 6 | 0 | 0 - 15.2 |  |  |
| *Scolopax rusticola* | 1 | 7 | 0 | 0 - 13.1 |  |  |
| *Spizaetus nipalensis* | 1 | 2 | 0 | 0 - 40.8 |  |  |
| *Strix uralensis* | 1 | 1 | 0 | 0 - 69 |  |  |
| *Struthio camelus* | 1 | 1 | 0 | 0 - 69 |  |  |
| *Turdus naumanii* | 1 | 1 | 0 | 0 - 69 |  |  |
| *Tyto alba* | 1 | 1 | 0 | 0 - 69 |  |  |
| *Upupa epops* | 1 | 1 | 0 | 0 - 69 |  |  |
| **Order** |  |  |  |  |  | p=0.8186 |
| *Accipitriformes* | 3 | 39 | 0 | 0 - 2.4 | 0 |  |
| *Anseriformes* | 2 | 31 | 0 | 0 - 3.1 | 0 |  |
| *Bucerotiformes* | 1 | 1 | 0 | 0 - 69 |  |  |
| *Charadriiformes* | 1 | 7 | 0 | 0 - 13.1 |  |  |
| *Ciconiformes* | 1 | 2 | 0 | 0 - 40.8 |  |  |
| *Falconiformes* | 3 | 28 | 18.3 | 0 - 83 | 89.1 |  |
| *Galliformes* | 3 | 380 | 0.7 | 0 - 6.3 | 88 |  |
| *Gruiformes* | 2 | 11 | 0 | 0 - 8.5 | 0 |  |
| *Passeriformes* | 2 | 111 | 3.5 | 0.9 - 7.7 | 0 |  |
| *Pelecaniformes* | 1 | 12 | 0 | 0 - 7.8 |  |  |
| *Phoenicopteriformes* | 1 | 2 | 0 | 0 - 40.8 |  |  |
| *Piciformes* | 1 | 1 | 0 | 0 - 69 |  |  |
| *Psittaciformes* | 1 | 12 | 0 | 0 - 7.8 |  |  |
| *Strigiformes* | 2 | 2 | 0 | 0 - 40.8 | 0 |  |
| *Struthioniformes* | 1 | 1 | 0 | 0 - 69 |  |  |
| **Wild versus captive** |  |  |  |  |  | p=0.7714 |
| Captive | 3 | 65 | 1.94 | 0 - 15.9 | 74.8 |  |
| Wild | 5 | 575 | 0.9 | 0 - 3.9 | 77.8 |  |
